# Electronic Health Record Based Algorithm to Identify Patients with Autism Spectrum Disorder

**DOI:** 10.1371/journal.pone.0159621

**Published:** 2016-07-29

**Authors:** Todd Lingren, Pei Chen, Joseph Bochenek, Finale Doshi-Velez, Patty Manning-Courtney, Julie Bickel, Leah Wildenger Welchons, Judy Reinhold, Nicole Bing, Yizhao Ni, William Barbaresi, Frank Mentch, Melissa Basford, Joshua Denny, Lyam Vazquez, Cassandra Perry, Bahram Namjou, Haijun Qiu, John Connolly, Debra Abrams, Ingrid A. Holm, Beth A. Cobb, Nataline Lingren, Imre Solti, Hakon Hakonarson, Isaac S. Kohane, John Harley, Guergana Savova

**Affiliations:** 1 Cincinnati Children's Hospital Medical Center, Division of Biomedical Informatics, Cincinnati, Ohio, United States of America; 2 Boston Children's Hospital, Center for Systems Biology, Boston, Massachusetts, United States of America; 3 Vanderbilt University School of Medicine, Biomedical Informatics, Nashville, Tennessee, United States of America; 4 Harvard Medical School, Center for Biomedical Informatics, Boston, Massachusetts, United States of America; 5 University of Cincinnati, Department of Pediatrics, Cincinnati, Ohio, United States of America; 6 Cincinnati Children’s Hospital Medical Center, Division of Developmental and Behavioral Pediatrics, Cincinnati, Ohio, United States of America; 7 Boston Children's Hospital, Pediatrics, Boston, Massachusetts, United States of America; 8 Boston Children's Hospital, Developmental Medicine, Boston, Massachusetts, United States of America; 9 Boston Children's Hospital, Neurology and Center for Communication Enhancement, Boston, Massachusetts, United States of America; 10 Children's Hospital Boston, Division of Medicine, Boston, Massachusetts, United States of America; 11 Children's Hospital of Philadelphia, Center for Applied Genomics, Philadelphia, Pennsylvania, United States of America; 12 Vanderbilt University Medical Center, Vanderbilt Institute for Clinical and Translational Research, Nashville, Tennessee, United States of America; 13 Boston Children's Hospital, Division of Genetics and Genomics, Boston, Massachusetts, United States of America; 14 Cincinnati Children's Hospital Medical Center, Center for Autoimmune Genomics and Etiology, Cincinnati, Ohio, United States of America; 15 University of Cincinnati, College of Medicine, Cincinnati, Ohio, United States of America; 16 Harvard Medical School, Pediatrics, Boston, Massachusetts, United States of America; 17 Boston Children's Hospital, Manton Center for Orphan Disease Research, Boston, Massachusetts, United States of America; 18 Cincinnati Children’s Hospital Medical Center, Emergency Medicine, Cincinnati, Ohio, United States of America; 19 Perelman School of Medicine, Pediatrics, Philadelphia, Pennsylvania, United States of America; 20 Boston Children's Hospital, Children’s Hospital Informatics Program, Boston, Massachusetts, United States of America; 21 United States Department of Veterans Affairs Medical Center, Medicine, Cincinnati, Ohio, United States of America; University of Illinois-Chicago, UNITED STATES

## Abstract

**Objective:**

Cohort selection is challenging for large-scale electronic health record (EHR) analyses, as International Classification of Diseases 9^th^ edition (ICD-9) diagnostic codes are notoriously unreliable disease predictors. Our objective was to develop, evaluate, and validate an automated algorithm for determining an Autism Spectrum Disorder (ASD) patient cohort from EHR. We demonstrate its utility via the largest investigation to date of the co-occurrence patterns of medical comorbidities in ASD.

**Methods:**

We extracted ICD-9 codes and concepts derived from the clinical notes. A gold standard patient set was labeled by clinicians at Boston Children’s Hospital (BCH) (N = 150) and Cincinnati Children’s Hospital and Medical Center (CCHMC) (N = 152). Two algorithms were created: (1) rule-based implementing the ASD criteria from Diagnostic and Statistical Manual of Mental Diseases 4^th^ edition, (2) predictive classifier. The positive predictive values (PPV) achieved by these algorithms were compared to an ICD-9 code baseline. We clustered the patients based on grouped ICD-9 codes and evaluated subgroups.

**Results:**

The rule-based algorithm produced the best PPV: (a) BCH: 0.885 vs. 0.273 (baseline); (b) CCHMC: 0.840 vs. 0.645 (baseline); (c) combined: 0.864 vs. 0.460 (baseline). A validation at Children’s Hospital of Philadelphia yielded 0.848 (PPV). Clustering analyses of comorbidities on the three-site large cohort (N = 20,658 ASD patients) identified psychiatric, developmental, and seizure disorder clusters.

**Conclusions:**

In a large cross-institutional cohort, co-occurrence patterns of comorbidities in ASDs provide further hypothetical evidence for distinct courses in ASD. The proposed automated algorithms for cohort selection open avenues for other large-scale EHR studies and individualized treatment of ASD.

## Introduction

With the prevalence of Autism Spectrum Disorders (ASD) at 1 in 68 children under the age of 8 years,[1] understanding the distinct clinical courses among patients with ASD is of great clinical relevance. In particular, increasing attention has been given to the comorbidity patterns of patients with ASD. Being able to effectively subcategorize patients with ASD has broad implications. A characterization of clinical courses can assist in risk stratification for future complications and inform more promising treatment directions. Dividing patient cohorts into more homogeneous subpopulations is also the first step toward more powerful genomic and molecular studies that can lead toward a better understanding of the etiologies involved in ASD.

Unfortunately, most studies to date that have examined the comorbidity patterns in ASD have been limited to smaller cohorts, as medical experts are needed to either query the patients directly or review their electronic health records (EHRs). These smaller studies do not have the statistical power to discover correlations across a large number of potentially relevant comorbidities.[2–6]

Studies based on large-scale EHR analysis have the potential to discover more complex relationships, such as clusters, among patients with ASD. For example, a recent study found distinct phenotypic clusters across a broad range of comorbidities using only diagnostic codes from the EHR.[7] The Electronic Medical Records and Genomics (eMERGE) Network[8] is a national consortium to study phenotype-genotype associations derived through large-scale high-throughput computing.[9–15] Similar computational approaches to cohort-extraction algorithms have proven successful by other major initiatives such as the Pharmacogenomics Research Network (PGRN)[16] and Informatics for Integrating Biology and the Bedside (i2b2).[17–19] The discipline of Electronic Health Record Driven Genomic Research (EDGR) is flourishing and has been reviewed in the mainstream genetics literature, a recognition that this domain of genomic research has come into its own.[20–21]

However, given the large numbers of patients involved, it is resource intensive to manually validate that each individual in the cohort actually has ASD via evaluation or chart review. The lack of a validated cohort limits what can be inferred from such large-scale studies. For example, the disease trajectories of patients who have simply been evaluated for ASD may be mixed with patients who have ASD. Similarly, genetic and molecular characterizations may be incorrect if the cohort is too impure. To reliably harness the power of large-scale, multi-institutional EHR data, we must be able to perform high-fidelity cohort-selection in an automated fashion.

The objective of this study is to employ the EHRs of multiple institutions to advance the study of ASD by 1) developing an automated algorithm for extracting cohorts and 2) examining the co-occurrence patterns of comorbidities associated with patients with ASD. Our algorithm is an important contribution in itself for future EHR-based studies of ASD; it provides a method for increasing the power and lowering the cost of cohort identification for genomic (or other clinical investigation) studies of ASD. Reproducing comorbidity associations in this cohort lends additional support to the subgroups of ASD found in both smaller studies with less statistical power and larger studies without validated cohorts.

## Patients and Methods

### Patients

**Fig 1:**
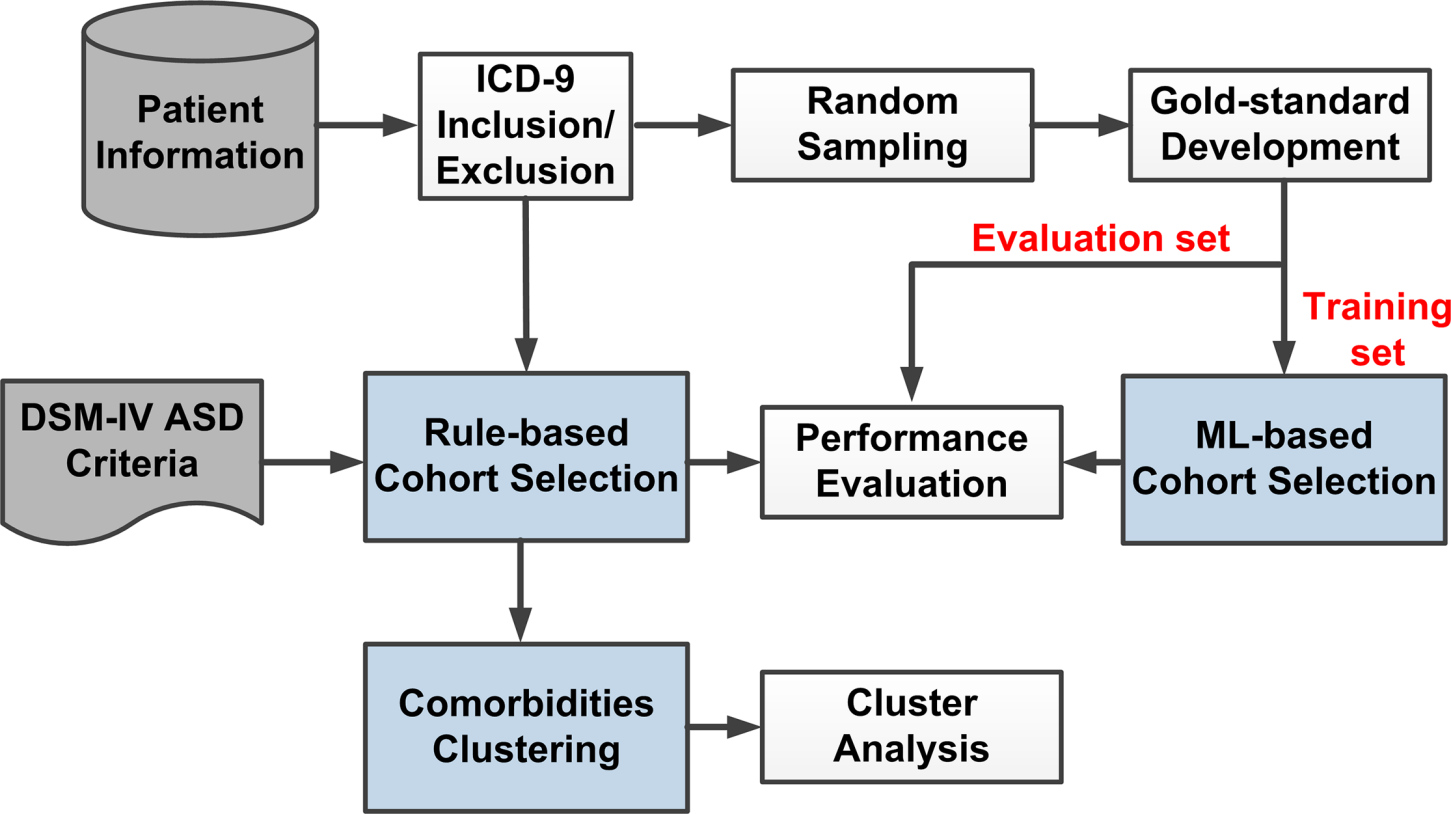
ASD Algorithm Project Overview. ASD–Autism Spectrum Disorder; ICD-9 –International Classification of Diseases 9^th^ edition; DSM IV–Diagnostic and Statistical Manual of Mental Diseases 4^th^ edition; ML—machine learning

The initial cohort consisted of all patients with International Classification of Diseases 9^th^ edition (ICD-9) diagnosis codes of 299.0, 299.80, 299.9 (Autism, Asperger’s, Pervasive Developmental Disorder Not Otherwise Specified (PDD-NOS), respectively) from the Boston Children’s Hospital (BCH) and Cincinnati Children’s Hospital Medical Center (CCHMC) EHR databases (14,758 and 4,229 patients, respectively) (Fig 1, ICD-9 Inclusion). Patients with diagnosis codes relating to Rett Syndrome, Childhood Disintegrative Disorder, Schizophrenia, Tuberous Sclerosis, and Fragile X Syndrome as well as severe Intellectual Disability (cognitively delayed or IQ < 40) or psychiatric illnesses (e.g. Bipolar Disorder, Schizophrenia) were excluded. The Institutional Review Board at each institution involved (Cincinnati Children's Hospital Medical Center, Boston Children's Hospital, Children's Hospital of Philadelphia, Vanderbilt University Medical Center) approved protocols using retrospective human subject data from electronic health records for this study. The waiver of consent was granted by the respective IRBs due to the nature of the retrospective study.

#### Gold standard set

The initial cohort, selected as described above, still contains false positive cases, patients who have an ICD-9 code in their medical record, but do not meet the clinical standard for diagnosis of ASD. For example, the ICD-9 code may have been used for billing purposes, but the ASD diagnosis was ruled out during clinical evaluation. From this initial ICD-9 screened cohort (BCH: 14,758, CCHMC: 4,229), 302 patients (150, 152, respectively) were randomly selected for gold standard chart review (see Fig 1, Random Sampling). The clinical notes of each patient were manually reviewed by clinicians to create a gold standard set of ASD diagnoses. The clinicians employed in double-annotated chart review are experienced in the clinical practice of ASD diagnosis (at BCH, one developmental-behavioral pediatrician (JB) and one psychologist (LWW); at CCHMC, one psychologist (NB), one nurse practitioner (JR) and one developmental-behavioral pediatrician (PMC)). There are four labels in the gold standard ASD diagnoses: “yes” indicates an ASD case; “no” label is a non-case; “maybe” is an uncertain case, some evidence for case; “unknown” is an indeterminate status, with insufficient information to make a decision relative to case status. After adjudication, ASD cases made up 44% of the gold standard (60% of CCHMC set, 26% of BCH set), 10% were “no” (16% CCHMC, 5% BCH), 36% were “maybe” (18% CCHMC, 55% BCH), and 10% were “unknown” (6% CCHMC, 13% BCH). After an initial training set of 20 patients at BCH and 40 patients at CCHMC, inter annotator agreement (IAA) was calculated using a pair-wise F-measure. IAA averaged among the three clinicians at CCHMC was 0.969 for ASD cases, 0.896 for “no”, 0.883 for “maybe”, and 0.75 for unknown. IAA at BCH was similar, 0.927 for “yes”, 0.625 for “no”, 0.86 for “maybe”, and 0.75 for “unknown”.

#### Inputs to EHR algorithms

Just as clinicians find the clinical notes critical for establishing the presence of ASD in their chart review, the clinical note was also an important source of our automated cohort selection algorithm. Specifically, the clinical notes from the initial cohort patients were transformed into a vector of concept unique identifiers (CUIs; e.g., C2675043 is the code for “Limited social interactions”) from the Unified Medical Language System (UMLS)[22] using the Apache cTAKES[23–24] natural language processing system. The default cTAKES dictionary (UMLS SNOMED-CT and RxNORM pruned by semantic types for Diseases/Disorders, Signs/Symptoms, Anatomical Sites, Medications and Procedures) was enriched with the terms from the Barbaresi list (S1 Table)[25]. If a term from the Barbaresi list was not represented in the UMLS, we created a project-specific code, for example there is no UMLS code for “stereotypical utterances” and we created CHIP1000204 (of note, all ASD codes not represented in the UMLS are preceded by CHIP (Children’s Hospital Informatics Program). cTAKES implements a full stack of NLP modules including part-of-speech tagger, parsers, relation discovery modules, as well as attribute identification modules (such as negation, uncertainty, subject). All clinical notes were processed through cTAKES and converted into concept vectors. For example, the sentence “patient diagnosed with ASD and inflammatory bowel disease” is represented by two CUIs: C1510586 (for ASD) and C0021390 (for inflammatory bowel disease) which in turn become the vector representation for this sentence. cTAKES employs a dictionary lookup algorithm with a sliding window to allow for term variations. The purpose of mapping the notes to CUIs was to ensure the portability of the algorithm across sites and take into account documentation differences for common symptoms. The average patient vector length was 344 CUIs (CCHMC, median 302) and 627 CUIs (BCH, median 484). These vectors formed the input to our two different automated cohort-selection algorithms.

#### Rule-based cohort selection

**Fig 2:**
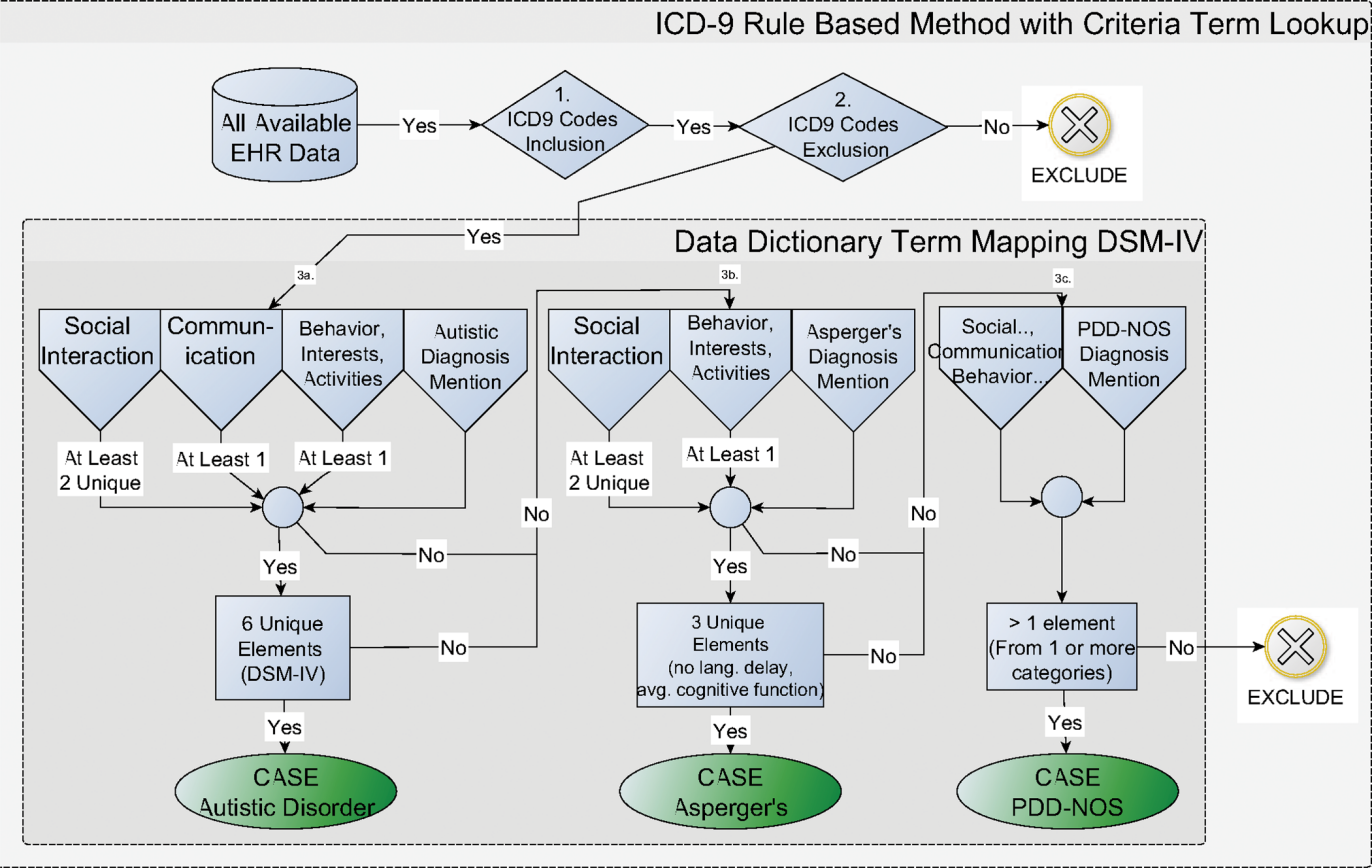
ASD Rule-based Algorithm. ASD–Autism Spectrum Disorder; EHR–Electronic Health Records; ICD-9 –International Classification of Diseases 9^th^ edition; DSM IV–Diagnostic and Statistical Manual of Mental Diseases 4^th^ edition; PDD-NOS–pervasive developmental disorder not otherwise specified; sections 3a., 3b., 3c. refer to DSM IV ASD classification for Autism, Asperger’s and PDD-NOS, respectively

For the rule-based algorithm (Fig 1, Rule-based Cohort Selection), a glossary of ASD symptoms[25] was mapped to UMLS CUIs by two physicians using UMLS Terminology Services.[26] The glossary contains a comprehensive list of descriptors corresponding to Diagnostic and Statistical Manual of Mental Diseases 4^th^ edition (DSM-IV) diagnostic criteria for ASD. Under the guidance of a clinician we manually mapped CUIs to terms from the glossary (see S1 Table). Fig 2 represents the complete rule-based algorithm. Steps 1 and 2 represent the initial cohort selection described in the Patients section. Step 3 (a, b, c) denote the three criteria counting steps, based on the DSM-IV symptom criteria for Autism, Asperger’s Syndrome, and PDD-NOS. The algorithm counted mentions of symptoms, represented by CUIs in the patient vector. Specifically, if the patient had at least 6 unique symptoms (at least two from Social Interaction, at least one from Communication and at least one from Behavior, Interests, and Activities), then the patient was determined to be a positive case for ASD. The three parts of step 3 are sequential. If a patient did not meet the criteria for Autistic Disorder (3a), the symptoms were assessed for the Asperger’s Syndrome criteria (3b). If the patient did not meet the threshold defined by Asperger’s Syndrome, the symptoms were compared to the criteria for PDD-NOS. If the patient did not meet the criteria for any of the diagnoses in 3a-c, that patient was considered a non-case. In this way, the rule-based algorithm implements the DSM-IV criteria. While the output of the rule-based algorithm includes sub-classes of ASD, for the purposes of evaluation and comparison with the machine learning algorithm all sub-classes of ASD are considered positive for ASD case. The text-based pseudocode for the rule-based algorithm is described in S1 File. The rule-based algorithm was validated at another eMERGE pediatric site, The Children’s Hospital of Philadelphia (CHOP). In addition, a fourth eMERGE site, Vanderbilt University Medical Center (VUMC), provided data and analysis for comorbidity clustering, described below.

#### Machine-learning cohort selection

**Fig 3:**
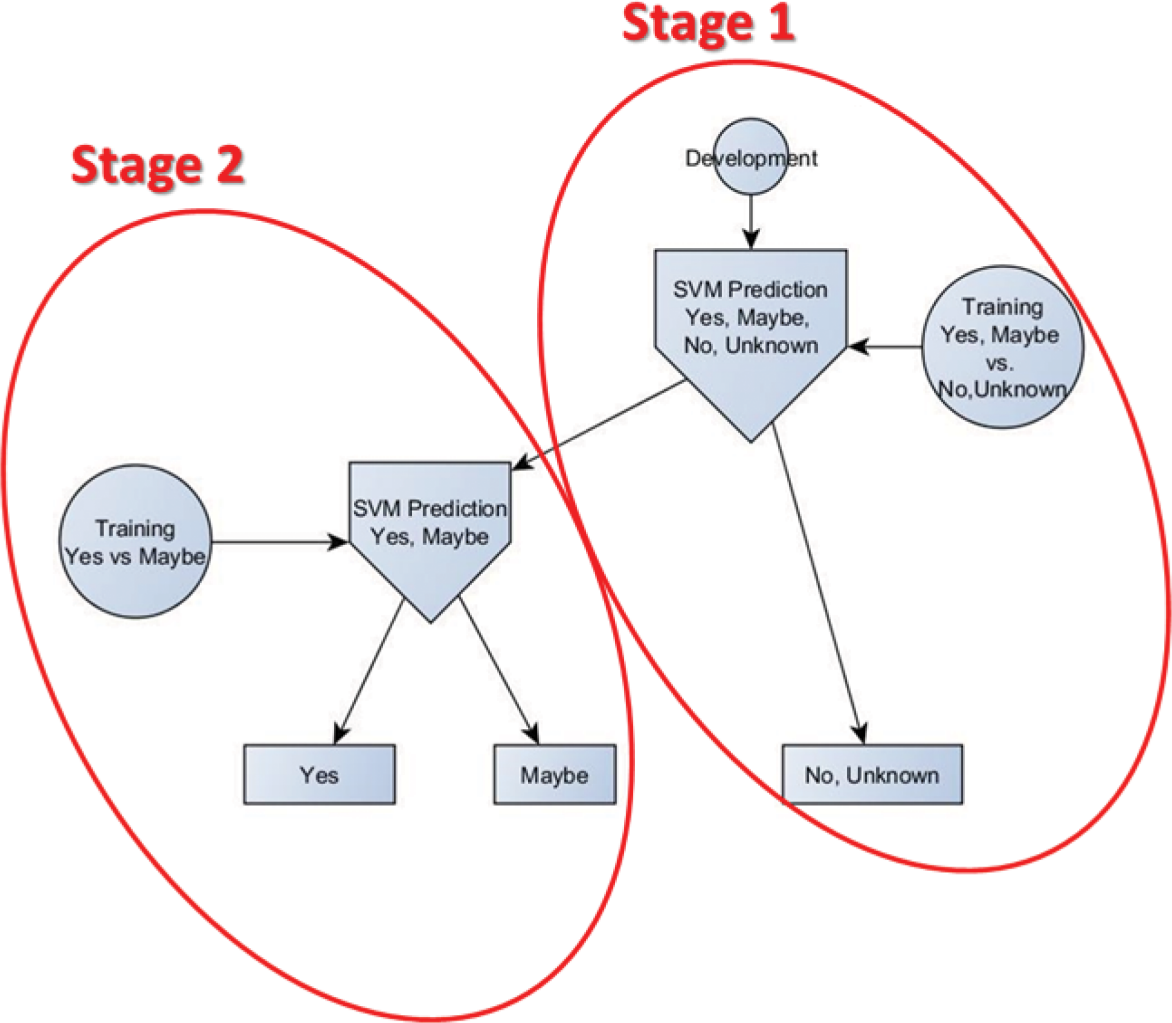
SVM-based Machine Learning Prediction System. SVM–Support Vector Machines.

We used the gold standard data set to derive an automated algorithm using machine learning to compare the rule-based approach (Fig 1, ML-based Cohort Selection). For developing the machine learning cohort selection algorithm, each site’s data were split up into groups to select 60% for training, 20% for development and 20% for testing/evaluation (see Table 1 for the distribution). We implemented the training-development-testing validation instead of k-fold cross-validation for two reasons: 1) the validation scheme could be easily adopted by different sites in the eMERGE network [27,28] and 2) the simplified setup enabled combining data from BCH and/or CCHMC in different training-testing experiments. Classifications were made using a two-stage Support Vector Machines (SVM)[29] system (Fig 3). The first stage differentiated patients that were either “yes” or “maybe” from patients that had “no” or “unknown” ASDs. The second stage was used to further differentiate the “yes” and “maybe” patients. The output of the first stage and the output of the second stage was then evaluated against the gold standard labels (Table 2, parts A and B, respectively). The SVM models were trained on the training set, tuned on the development set and evaluated on the test set. SVM is an effective classification method, but it does not directly do feature pruning (regularization) although the features are ranked. Irrelevant features in the feature vectors would inevitably cause inaccuracy in similarity measurement (i.e. kernels) between samples, would decrease the generalizability of the models and increase the overall computational time. Hence, similar to other work [29–32] we combined SVM with feature selection to enhance the performance. First, feature selection was performed to reduce the dimensionality of the patient vectors. Specifically, we calculated the chi square value of each feature in each vector set to rank the features by significance. The best number of features was selected based on the model performance on the development set as described in the Results section.

**Table 1:** Distribution of patients

	Training	Development	Test	Total
BCH	95	27	28	150
CCHMC	87	34	31	152
Combined	182	61	59	302

BCH–Boston Children’s Hospital; CCHMC–Cincinnati Children’s Hospital and Medical Center.

**Table 2:** Best Machine Learning Results on Test Set.

**A: One Stage–Yes, Maybe vs. No, Unknown**					
**Training Set**	**Test Set**	**Precision/PPV**	**Recall/Sensitivity**	**F1-Measure**	**Area under ROC Curve**
1st stage: Combined	BCH	0.726	0.852	0.784	0.533
1st stage: CCHMC	CCHMC	0.66	0.813	0.728	0.545
1st stage: Combined	Combined	0.864	0.836	0.785	0.583
**B: Two Stage, Yes vs. No, Maybe, Unknown**					
**Training Set**	**Test Set**	**Precision/PPV**	**Recall/Sensitivity**	**F1-Measure**	**Area under ROC Curve**
2^nd^ stage: Combined	BCH	0.780	0.783	0.780	0.762
2^nd^ stage: Combined	CCHMC	0.799	0.769	0.780	0.733
2^nd^ stage: Combined	Combined	0.786	0.769	0.761	0.770

BCH–Boston Children’s Hospital; CCHMC–Cincinnati Children’s Hospital and Medical Center; PPV–positive predictive value; ROC–Receivers Operator Characteristic.

Within the eMERGE project, the metric of greatest interest for phenotyping is Precision/PPV on the “yes” category.[33–35] This is motivated by the expectation that the automatically discovered cohort will be of high purity to be useful for genotyping studies. Some of the cases can be lost because of the impact on sensitivity, but patients labeled as cases are expected to be of high fidelity. Statistical power for analysis purposes is gained by combining the cases from all sites. Accordingly, parameters were tuned in development to maximize PPV.

#### Cohort quality validation

The performance of the two algorithms was evaluated in precision (PPV), recall (or sensitivity) and F-measure (the harmonic mean of precision and recall, (2*precision*recall) / (precision+recall)).

### Comorbidities Clustering

There is evidence that the clinical manifestations of common neurodevelopmental disorders such as ASD do not always correspond to diagnostic definitions.[36] A data-driven method for exploring this hypothesis is to consider the comorbidity pattern for patients who are diagnosed with ASD. This approach allows us to mine existing EHR data for insights to refine disease categorization. For example, a previous study showed that patients with ASD were significantly more likely to be diagnosed with certain comorbidities including Epilepsy, Schizophrenia, Inflammatory Bowel Disease, and Cranial Abnormalities, when compared to the general population [7,37]. Another study used an automated clustering algorithm to demonstrate the existence of clusters within ASD patients when patients are characterized by their set of comorbidities. The objective of clustering is to uncover patterns in data that are not apparent with traditional analysis due to the size and complexity of the dataset. The clusters found in [7] were characterized by other psychiatric disorders, seizures, and gastrointestinal disorders, in other words, many of the same comorbidities previously found to be of greater prevalence in patients with ASD.

We perform a study similar to the one performed in [7] but with several improvements: 1) Patients are selected using the algorithm presented in this paper, providing a cleaner sample of ASD patients 2) we extend the analysis to multiple institutions, thus using a significantly larger dataset, 3) We compare the results of several different clustering and visualization algorithms. This serves not only as a validation of the original study, but to facilitate the application of clustering to studies which require greater statistical power and larger sample sizes, such as targeted genetic association studies.

We clustered comorbidities based on the disease codes of the automatically mined ASD patient cohort (Fig 1, Comorbidities Clustering). We included a dataset from a third institution, VUMC, to evaluate the generalizability of our results. We first identified patients from BCH, CCHMC and VUMC using the best performing algorithm (rule-based) on the three patient sets from among 14,758 BCH patients, 4,229 CCHMC patients and 6,482 VUMC patients. The rule-based algorithm identified 87.7% of BCH patients (12,949), 70.7% of CCHMC patients (2,988), and 72.8% of VU patients (4,721) as ASD cases. We pre-processed the data by converting the patients’ ICD-9 codes into Phenotype Wide Association Study (PheWAS) categories[38] and excluded PheWAS categories present in less than 0.5% of patients. We then performed clustering on the patients represented by the resulting PheWAS code vectors using the k-means algorithm.[39] In k-means clustering, we varied the number of clusters between 2 and 20 in order to find the clustering with the highest silhouette coefficient. We compared these results to two additional clustering algorithms (DBSCAN and Agglomerative Clustering).[40–41] Both algorithms were either found to have inferior cluster separation or to yield clusters with little discernable meaning. Clusters were characterized by the relative prevalence of PheWAS codes in each cluster as well as the percentage of all patients with each code in the cluster.

## Results

### Automated Cohort Selection Algorithms

The rule-based results were evaluated by including the gold-standard “yes” and “maybe” labels as positive for case (Table 3, see Fig 1, Performance Evaluation). The evaluation set for the rule-based results consisted of the entire 302 patients described above. None of these patients were used in developing the rule-based algorithm which was an implementation of the DSM-IV diagnosing criteria. The output of the rule-based algorithm included four possibilities (see Fig 2), grouped together by predicted case (ASD, Asperger’s, PDD-NOS) or predicted non-case (exclude). Because there was a high correlation between gold standard “maybe” label and system-predicted PDD-NOS (case), the evaluation of the rule-based results includes the “maybe” patients as a true case.

**Table 3:** Rule Based Results

Evaluation Set	Precision/PPV	Recall/Sensitivity	F1-Measure	Area under ROC Curve
BCH	0.885	0.891 (14)	0.888	0.642
CCHMC	0.840	0.622 (48)	0.715	0.599
Combined	0.866	0.758 (62)	0.808	0.579
CHOP	0.849	0.737 (10)	0.788	0.659
(independent validation on 50 patients)				

BCH–Boston Children’s Hospital; CCHMC–Cincinnati Children’s Hospital and Medical Center; CHOP–Children’s Hospital of Philadelphia; PPV–positive predictive value; ROC–Receivers Operator Characteristic.

The machine learning based results are presented in Table 2 (see Fig 1, Performance Evaluation). Using the best performing machine learning model tuned on the development sets, the results of evaluation of Stage 1 and Stage 2 (see Fig 3) are presented in Table 2. In alignment with the rule based algorithm, in Stage 1 we considered “yes” and “maybe” gold standard labels as case and “no” and “unknown” to be non-case. Part A of Table 2 is the performance the Stage 1. The second stage further classified “yes” and “maybe” labels from the positive prediction of Stage 1. Table 2, Part B presents the combined performance of both Stage 1 and Stage 2, where the gold standard label “yes” was case and the rest was non-case.

**Fig 4:**
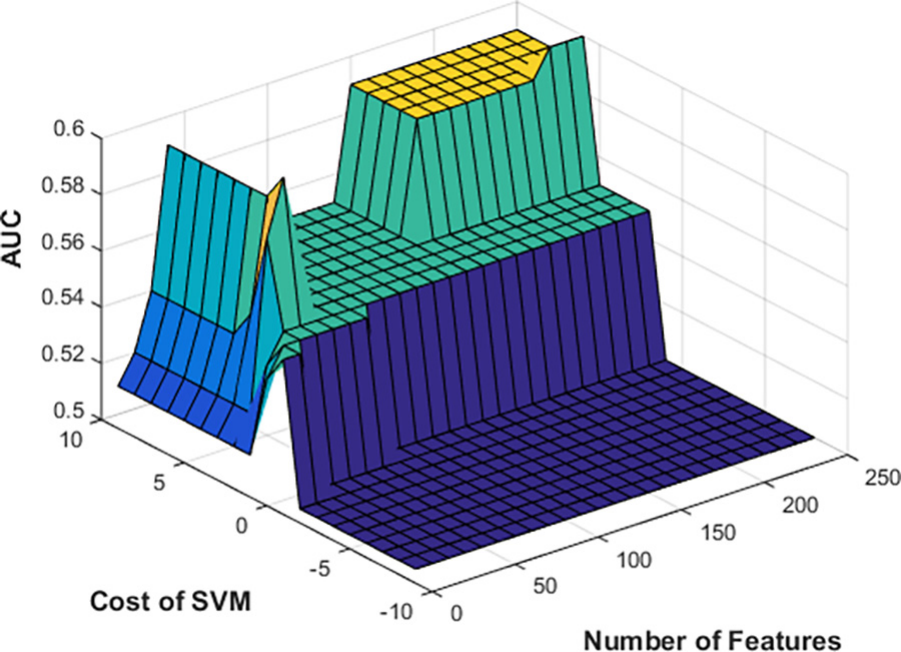
SVM Grid Search on development set, using features and cost parameters.

To tune the performance of the machine learning algorithm, we used a grid search method to examine different feature sets and cost parameters. We selected the best features and parameters on the development set, based upon the PPV performance. The grid search for the development set is depicted in Fig 4. The cost axis is showing in log scale (values used were from 2^−10^ to 2^10^). After examining the prediction performance on the development set, we set an upper empirical bound on the number of features, given that the results plateaued at 250. Performance in Fig 4 is shown in area under the receiver operator curve (AUC). This measure is also shown in Tables 2 and 3 for comparison.

The results presented are on the test set using those selected features and cost parameters. The number of best performing features and feature sets varied between sites. In Stage 1 (Table 2A) the combined feature set achieved the best performing score on the development set when used as training data for BCH and the Combined data set. In both cases, 40 features were sufficient to achieve a test PPV of 0.726 and 0.864, respectively. For the CCHMC data set, the best performance was reached on the development set with 210 features from CCHMC data as training. The model suffered from some overtraining on the development set and only achieved a test set PPV of 0.66 (development PPV was 0.894). In Stage 2 (Table 2B), where the goal was to separate the “yes” case from the “maybe” instances, all three data sets performed best when using the combined data set as training. CCHMC and Combined data sets performed best on the development set with 200 features; BCH used the top 70 features. All three development sets had AUC values at or above 0.95, while the test AUC dropped to the 0.73 to 0.77 range.

Baseline comparison, shown in Table 4, was done against an algorithm which uses only relevant ICD-9 codes to retrieve patients as described in the Methods section (Fig 1, ICD-9 Code Inclusion/Exclusion). Recall/sensitivity cannot be computed for the baseline because no patients were evaluated if they did not match the ICD-9 criteria (e.g. no patients without ASD ICD-9 codes exist in cohort).

**Table 4:** Baseline Results (ICD-9 codes) on Test Set.

Test Set	Precision/PPV
BCH	0.273
CCHMC	0.645
Combined	0.460

BCH–Boston Children’s Hospital; CCHMC–Cincinnati Children’s Hospital and Medical Center; PPV–positive predictive value.

The best performing algorithm (the rule-based algorithm) was validated by another eMERGE site–CHOP. They implemented the algorithm and performed an independent chart review of 50 patients, with a PPV of 84.9% and Sensitivity of 73.7% (Table 3).

### Comorbidities Clustering

Clusters are characterized by the prevalence of PheWAS codes in each cluster. However, in order to compare cluster results with previous studies and to better visualize the results, we grouped PheWAS codes according to comorbidity groups for Seizures, Psychiatric, Auditory, Developmental, GI Disorders, and Cardiac Disorders. The clustering was performed on the patients from each site: BCH, CCHMC, and VUMC, identified through the best performing algorithm (the rule-based, see Fig 1, Comorbidities Clustering). By comparing multiple sites, we evaluated the possibility that clusters are a result of statistical variation, idiosyncrasies in coding practices at a particular institution, or other site-based effects.

**Fig 5:**
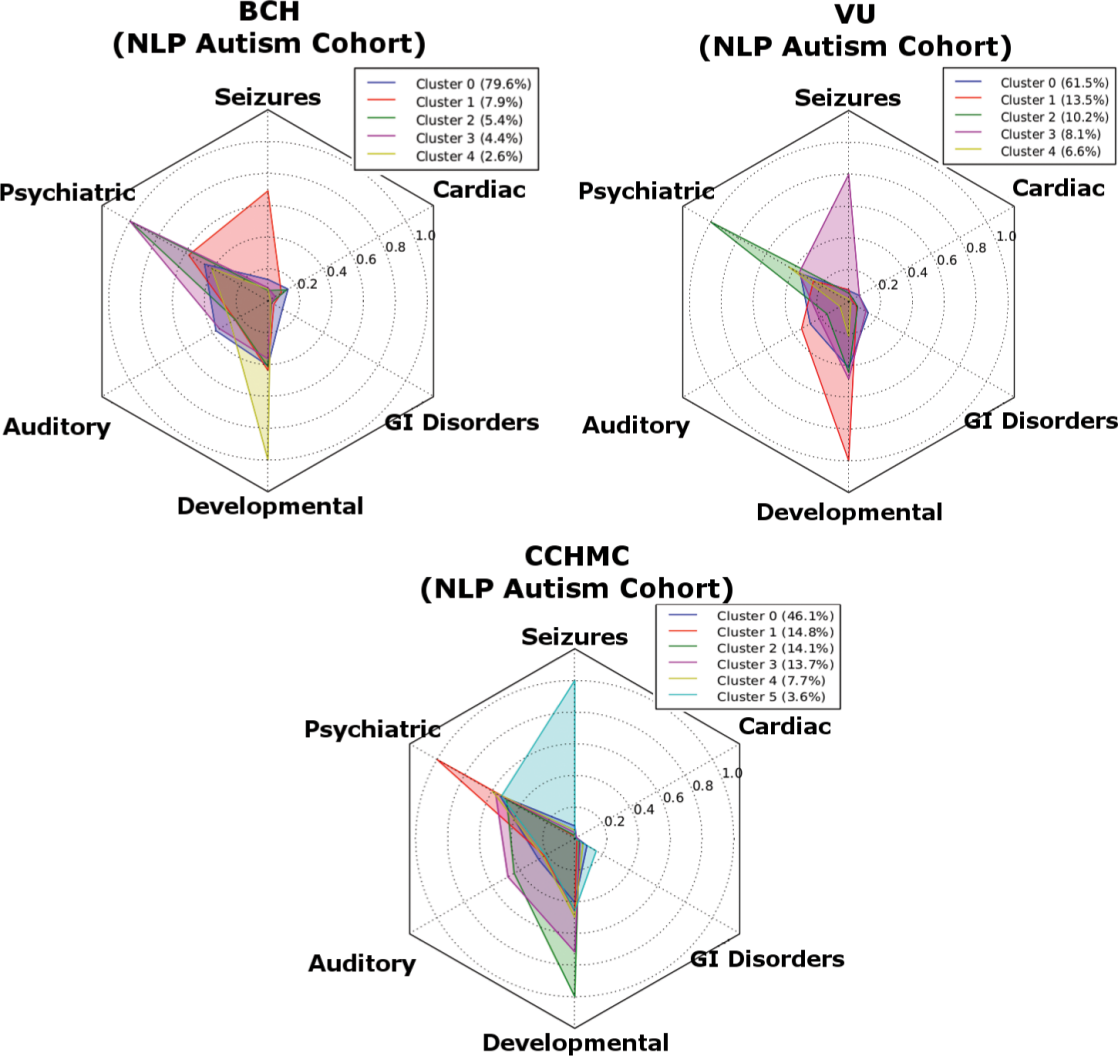
Comparison of relative prevalence of primary co-morbidity categories for clusters of NLP rule-based patients for BCH, and CCHMC and VUMC. BCH–Boston Children’s Hospital; CCHMC–Cincinnati Children’s Hospital and Medical Center; VUMC–Vanderbilt University Medical Center.

We found that each institution exhibits a similar clustering pattern (Fig 5). In each set we identified three or four small clusters (5–20% of the overall set), and one large cluster. Each of the smaller clusters is dominated by one of several comorbidity categories: 1) psychiatric problems including anxiety disorder, hyperkinetic syndrome, obsessive compulsive disorder, and depression; 2) developmental disorders including dyslexia, lack of coordination, and various disorders of the ear, skin and other bodily systems; 3) epilepsy and recurrent seizure. The larger cluster, comprising 60–80% of the set, was not characterized by a high prevalence of any comorbidity or category of comorbidities.

**Fig 6:**
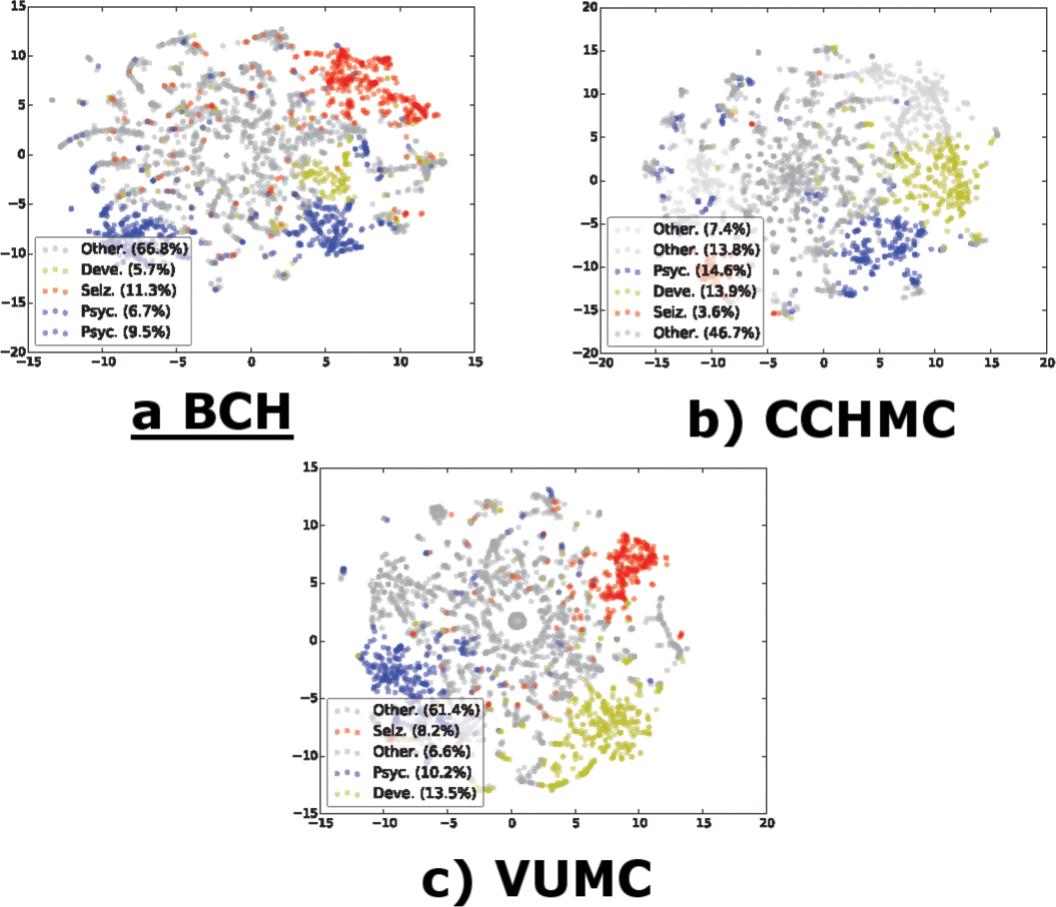
Dimensionality reduction using the t-SNE algorithm on PheWAS codes. Colors label clusters from the k-means algorithm. The clusters are labeled according to the comorbidity category with the highest relative prevalence for that cluster—duplicate labels appear when there is more than one cluster dominated by the same category. (t-distributed Stochastic Neighbor Embedding—t-SNE, Phenotype Wide Association Study–PheWAS, BCH–Boston Children’s Hospital; CCHMC–Cincinnati’s Children’s Hospital and Medical Center; VUMC–Vanderbilt University Medical Center, Deve.–Developmental Disorders, Seiz.–Seizure Disorders, Psych.—Psychological Disorders).

Since the patients were represented in high dimensionality (100–200 PheWAS codes), we used a dimensionality reduction algorithm to visualize the separation of the clusters. Dimensionality reduction provides a representation of the dataset in a reduced number of dimensions while maintaining a measurement of the separation between each data point. This provides a method to visualize the nature of the clustering pattern uncovered by the clustering algorithms. We used the t-distributed Stochastic Neighbor Embedding[42] to visualize the PheWAS code vectors in two dimensions. The results are shown in Fig 6. The visualization was independent of any of the automated unsupervised clustering algorithms and gives both validation and additional visualization insight in to the nature of the clusters uncovered by the clustering analysis (Fig 5). Patients with significant codes in the categories for psychiatric disorders, developmental disorders, and seizures are grouped together and distinct from the bulk of the patients, whereas most patients fall into a large cluster that cannot be further subcategorized using comorbidities.

## Discussion

Running analytical algorithms on large cohorts enabled detection of useful clinically distinct comorbidity subgroups of ASD. The cohort assembled from the three sites is the largest ASD cohort so far (20K+ patients). A limitation of the study is that the cohort does not represent a gold standard, since it was not feasible to perform chart review on all 20K+ patients. The results for the EHR-based ASD algorithm for the diagnosis component vary between institutions. The focus of the methods evaluation was PPV because an eMERGE goal is for genomic discovery of variants associated with phenotypes. A strong PPV, then, is preferred. The rule-based algorithm performed better on the BCH data (BCH, 0.885 PPV; CCHMC, 0.840 PPV), while the machine learning algorithm performed similarly at both sites (BCH, 0.780 PPV; CCHMC, 0.799 PPV). The disparate amount of data available as well as different ASD diagnostic models and documentation practices might explain the difference. The rule-based algorithm is sensitive to a signal of any amount (mentions of symptoms relevant to DSM-IV criteria). The BCH data has a longer term of data (since 2004), while the CCHMC data set represents patient encounters from January 2010—June 2013. Symptoms for case prediction are more likely to be present in the larger volume of BCH patient data. The baseline results for BCH are worse than for CCHMC (PPV = 0.273 and 0.625, respectively). A high number of gold standard ‘maybe’ patients were predicted at both sites to be PDD-NOS case by the rule-based algorithm case definition which is built on the DSM-IV criteria. Including these ‘maybes’ as positive cases in evaluation maintains the fidelity of the DSM-IV criteria as automatically applied to the EHR records of patients. The baseline results illustrate the importance of not relying only on ICD-9 codes for accurate phenotyping. Further, the similarity of the PPV between the rule-based and machine learning algorithms highlights the advantage that machine learning can provide over a potentially resource intensive knowledge-based algorithm. One limitation of the current data set is that the AUC values on the test set for either CCHMC or BCH did not exceed 0.55 in Stage 1 experiments, possibly indicating that the development set for one site is not large enough to provide a representative sample to examine a model. The similarity of the result from the machine-learning based algorithm, given that the best performing results came from a combined training set, indicates a potential for multi-site aggregation of data to improve predictive power.

Although Apache cTAKES is a state-of-the-art system, it is not a perfect system (as none of the NLP systems are perfect at this point of time). Error analyses points to inaccuracies due to (1) language variations, (2) world knowledge, (3) meta knowledge. The first category is represented by the many ways ASD behavior can be described. Although we use the Barbaresi list (S1 Table) which has been the gold standard for the ASD domain, we are likely not capturing the linguistic richness of expressing the many facets of ASD behavior. The second source of errors stems from world knowledge, for example the mention that the patient goes to a certain school is unequivocal evidence to the physician that the patient is highly likely to be autistic. This kind of world knowledge is not encoded in cTAKES and in general is a challenge for NLP systems. The last source of errors is in the importance of meta knowledge such as the department originating the ASD diagnosis. The department for an ASD diagnosis is not uniform among different institutions; some institutions have primary departments for such disorders, leading to a more confident assurance of diagnosis. We avoided encoding very specific heuristics such as physician names or departments which would greatly reduce the generalizability and portability of the algorithm.

Using conditional analyses, clustering, and dimensionality reduction, we explored and characterized the comorbidity structure of ASD patients at three institutions. The usefulness of this task is demonstrated in classifying subgroups of ASD patients based on their common comorbidities. We investigated the effect of the rule-based algorithm on comorbidity clustering. Our results provided replication and refinements of previous work done on comorbidity clustering, showing that there are clear subgroups in ASD patients represented by their clinical comorbidities. Most notably, we found that there were three conditionally independent clusters: one characterized by seizures; one characterized by psychiatric disorders; and one characterized by developmental delays. Other major comorbidities which were elevated in the ASD population were not found to be independent and did not result in separate clusters. The results replicate and refine ASD comorbidity studies reported previously on smaller cohorts, and extend these studies to multiple institutions. The two methods presented in this paper provide automated techniques to extract and refine ASD cohorts and provide complementary approaches to harness the EHR in order to increase the power of genomic studies of ASD. Future research is needed to identify successful treatment sets for each ASD subgroup, or comorbidity cluster. Applying the algorithm to cohorts which do not have ICD-9 code diagnoses of ASD could yield further insights into diagnosis and treatment. Further work would be useful in applying the clustering techniques in this paper to other complex disorders for assessment of morbidity, subtyping or treatment similar to previous work.[43–45]

## Conclusion

Cohort selection is a significant issue for large scale EHR analyses and reuse of such data may provide insights into causes, risks, and treatments for diseases such as ASD. This study provides a validated EHR-based natural language processing ASD prediction algorithm applied to a large multi-institutional cohort. We demonstrated feasibility of mining the EHR across multiple institutions with the same algorithm to generate a large cohort of ASD patients. Using that large cohort to study ASD comorbidities our research confirmed previous studies with smaller sample sizes that found several distinct co-morbidity clusters in ASD. Our algorithm allows for the automated creation of a high-fidelity cohort which opens avenues for other large scale EHR studies and may further the ability to research specific treatment courses based on comorbidity cluster as well as genetic and molecular characterizations. Comparing results from BCH and CCHMC indicates the benefit of a comprehensive longitudinal EHR and not relying on ICD-9 codes for phenotyping. Further research is recommended to identify successful treatment courses for each ASD subgroup or comorbidity cluster.

## Supporting Information

S1 File Rule-Based Algorithm Pseudocode. (DOC)

S1 Table CUI Symptom Mapping. (DOC)
