# Supplementary material for: Electronic Health Record Based Algorithm to Identify Patients with Autism Spectrum Disorder
Source: PLoS One. 2016 Jul 29;11(7):e0159621. doi: 10.1371/journal.pone.0159621 (PMC4966969; doi:10.1371/journal.pone.0159621)
Supplement: S1 File — (DOC) [file pone.0159621.s001.doc]

# S1 File – Rule-Based Algorithm Pseudocode

1. At least 1 inclusion ICD-9 codes for Autism, Asperger’s and PDD-NOS (299.0, 299.80,299.9)

YES -> Go to Step 2

No -> **EXCLUDE***

1. At least 1 exclusion ICD-9 codes : 299.1,295*,330.8,759.5,759.83

YES -> **EXCLUDE**

NO -> Go to Step 3

1. DSM-IV Symptom criteria (Reference CUI Symptom Mapping)

3a. DSM-IV (total at least 6 symptoms): At Least 2 unique from Social Interaction AND at least 1 from Communication AND at least 1 from Behavior, Interests and Activities

YES -> Autistic Disorder **CASE**

NO -> Go to step 3b.

3b. DSM-IV: At Least 2 unique from Social Interaction AND at least 1 from Behavior, Interests and Activities AND no language delay AND average cognitive function

YES -> Asperger’s Syndrome **CASE**

NO -> Go to Step 3c.

3c. DSM-IV: More than one symptom from 1 or more categories of Social Interaction OR Communication OR Behavior, Interests and Activities

YES-> PDD-NOS **CASE**

NO-> **EXCLUDE**

***** Algorithm termination points are in bold.
