# Supplementary material for: Electronic Health Record Based Algorithm to Identify Patients with Autism Spectrum Disorder
Source: PLoS One. 2016 Jul 29;11(7):e0159621. doi: 10.1371/journal.pone.0159621 (PMC4966969; doi:10.1371/journal.pone.0159621)
Supplement: S1 Table — (DOC) [file pone.0159621.s002.doc]

# S1 Table – CUI Symptom Mapping

| Asperger’s |  |  |
| --- | --- | --- |
| CUI* | TEXT | CODE |
| †C0236792 | Asperger’s | C0236792 |
| C1837434 | ASPERGER SYNDROME, SUSCEPTIBILITY TO, 3 | C1837434 |
| C1837646 | ASPERGER SYNDROME, SUSCEPTIBILITY TO, 1 | C1837646 |
| C1837697 | ASPERGER SYNDROME, SUSCEPTIBILITY TO, 2 | C1837697 |
| C1864961 | ASPERGER SYNDROME, SUSCEPTIBILITY TO, 4 | C1864961 |
| C1845334 | ASPERGER SYNDROME, X-LINKED, SUSCEPTIBILITY TO, 2 (finding) | C1845334 |
| C1845341 | ASPERGER SYNDROME, X-LINKED, SUSCEPTIBILITY TO, 1 (disorder) | C1845341 |
| C3151708 | ASPERGER SYNDROME, SUSCEPTIBILITY TO, X-LINKED 1 | C3151708 |
| C3151722 | ASPERGER SYNDROME, SUSCEPTIBILITY TO, X-LINKED 2 | C3151722 |
|  | | |
| Autism |  |  |
| CUI | TEXT | CODE |
| C0004352 | Autistic Disorder | C0004352 |
| C0175842 | Autistic Children | C0175842 |
| C0233639 | Autistic thinking | C0233639 |
| C0856975 | Autistic behavior | C0856975 |
| C1306579 | psychopathy; autistic | C1306579 |
| C1846135 | Autistic features | C1846135 |
| C0154446 | Infantile autism, current or active state | C0154446 |
| C0338986 | Atypical autism | C0338986 |
| C1389182 | mental retardation; autistic features | C1389182 |
| C1839793 | Autistic-like behavior | C1839793 |
| C1841787 | Autism or autistic-like condition | C1841787 |
| C1846042 | Autistic features (rare) | C1846042 |
| C2749029 | Autistic-like features | C2749029 |
| C3279328 | Autistic features (if left untreated) | C3279328 |
| C1298684 | Autistic spectrum disorder with isolated skills | C1298684 |
| C1845866 | Behavioral changes consistent with an autistic disorder | C1845866 |
| C2062756 | autistic disorder of childhood onset with full syndrome | C2062756 |
| C2062757 | autistic disorder of childhood onset with residual state | C2062757 |
| C3148934 | MENTAL RETARDATION WITH LANGUAGE IMPAIRMENT AND AUTISTIC FEATURES, 390-KB DE | C3148934 |
| C1510586 | Autism Spectrum Disorders | C1510586 |
| C3278858 | Autism spectrum disorder (less common) | C3278858 |
|  |  |  |
| PDD-NOS |  |  |
| CUI | TEXT | CODE |
| C0524528 | Pervasive development disorder | C0524528 |
| C0008074 | Child Development Disorders, Pervasive | C0008074 |
| C0349331 | Other pervasive developmental disorders | C0349331 |
| C0851877 | Pervasive developmental disorders NEC | C0851877 |
| C1839006 | Pervasive developmental disorder (CVS+) | C1839006 |
| C2062758 | atypical pervasive developmental disorder | C2062758 |
| C1456313 | Other specified pervasive developmental disorders | C1456313 |
| C2874989 | Pervasive and specific developmental disorders (F80-F89) | C2874989 |
|  |  |  |
| General |  |  |
| CUI | TEXT | CODE |
| C1445953 | moderate eye contact | C1445953 |
| C1445953 | eye contact fair | C1445953 |
| C1445953 | eye contact less than optimal | C1445953 |
| C1445953 | eye contact is present about 1/2 of time | C1445953 |
| C1445953 | sometimes making poor eye contact | C1445953 |
| C1445953 | intermittent eye contact | C1445953 |
| C1445953 | made intermittent eye contact | C1445953 |
| C1445953 | occasional brief eye contact | C1445953 |
| C1445953 | inconsistent eye contact | C1445953 |
| C1445953 | prefers to look at people out of corner of eye | C1445953 |
| C1445953 | eye contact was variable | C1445953 |
| C1445953 | eye contact mildly diminished | C1445953 |
| C1445953 | eye contact moderately reduced | C1445953 |
| C1445953 | decreased eye contact | C1445953 |
| C1445953 | diminished eye contact with examiner | C1445953 |
| C1445953 | eye contact of marginal quality | C1445953 |
| C1445953 | limited eye contact | C1445953 |
| C1445953 | little eye contact | C1445953 |
| C1445953 | makes little eye contact | C1445953 |
| C1445953 | eye contact fleeting | C1445953 |
| C1445953 | looked at me only fleetingly | C1445953 |
| C1445953 | he only provides a fleeting eye contact | C1445953 |
| C1445953 | eye contact only fleetingly achieved | C1445953 |
| C1445953 | eye contact difficult to obtain, tended to be fleeting | C1445953 |
| C1445953 | eye contact abnormal | C1445953 |
| C1445953 | eye contact is present only sporadically; exhibits social smile only sporadically | C1445953 |
| C1445953 | eye contact was marginal, usually fleeting, typified by lack of social acknowledgment | C1445953 |
| C1445953 | difficult to obtain or maintain eye contact | C1445953 |
| C1445953 | difficult to maintain eye contact | C1445953 |
| C1445953 | eye contact very difficult | C1445953 |
| C1445953 | rarely maintained eye contact | C1445953 |
| C1445953 | rarely makes eye contact | C1445953 |
| C1445953 | rarely establishes eye contact | C1445953 |
| C1445953 | he did not initiate eye contact | C1445953 |
| C1445953 | did not initiate any eye contact | C1445953 |
| C1445953 | avoided eye contact | C1445953 |
| C1445953 | avoided direct gaze | C1445953 |
| C1445953 | avoiding any eye contact | C1445953 |
| C1445953 | assiduously avoided any eye contact | C1445953 |
| C1445953 | he avoided eye contact with examiner | C1445953 |
| C1445953 | facial expressions constricted, and eye contact | C1445953 |
| C1445953 | trouble making eye contact | C1445953 |
| C1445953 | minimal eye contact | C1445953 |
| C1445953 | impairment in eye-to-eye gaze | C1445953 |
| C1445953 | does not make good eye contact | C1445953 |
| C1445953 | did not establish good eye contact | C1445953 |
| C1445953 | did not sustain eye contact | C1445953 |
| C1445953 | very little eye contact | C1445953 |
| C1445953 | very little eye contact with others | C1445953 |
| C1445953 | very limited eye contact | C1445953 |
| C1445953 | makes very little eye contact | C1445953 |
| C1445953 | makes scarce eye contact | C1445953 |
| C1445953 | poor eye contact | C1445953 |
| C1445953 | shows poor eye contact | C1445953 |
| C1445953 | very poor eye contact | C1445953 |
| C1445953 | eye contact very poor | C1445953 |
| C1445953 | makes very poor eye contact | C1445953 |
| C1445953 | eye contact is virtually nill | C1445953 |
| C1445953 | eye contact is observed 3 times during 45 minute interview | C1445953 |
| C1445953 | never looks you in the eye | C1445953 |
| C1445953 | does not establish eye contact | C1445953 |
| C1445953 | eye contact never established | C1445953 |
| C1445953 | lack of eye contact | C1445953 |
| C1445953 | he did not provide any eye contact | C1445953 |
| C1445953 | no eye contact with examiner | C1445953 |
| C1445953 | no eye contact | C1445953 |
| C1445953 | eye contact non-existent | C1445953 |
| C0813217 | flat facial expression | C0813217 |
| C0813217 | facial expression is lacking for any social interchange | C0813217 |
| C0813217 | no indication of affect in his face | C0813217 |
| C0813217 | expressionless face | C0813217 |
| C0813217 | lack of facial expression | C0813217 |
| C0813217 | mask-like facial expression | C0813217 |
| C0813217 | poverty of facial expression | C0813217 |
| C0566009 | non-verbal social skills are poor | C0566009 |
| C0566009 | abnormal non-verbal communication | C0566009 |
| C0566009 | no systematic, interactive non-verbal communication | C0566009 |
| C0566009 | does not use body posture to communicate | C0566009 |
| C0566009 | does not use gestures to communicate | C0566009 |
| C0566009 | difficulty using non-verbal communication | C0566009 |
| C2372111 | will not play with others | C2372111 |
| C2372111 | plays alone | C2372111 |
| C2372111 | doesn't play with anyone | C2372111 |
| C2372111 | plays by himself | C2372111 |
| C2372111 | solitary in his play | C2372111 |
| C2372111 | he only plays by himself | C2372111 |
| C2372111 | plays for long periods by himself | C2372111 |
| C2372111 | prefers to play alone | C2372111 |
| C2372111 | likes to play by himself | C2372111 |
| C2372111 | most of time he plays by himself | C2372111 |
| C2372111 | usually plays alone | C2372111 |
| C2372111 | does not play with other children | C2372111 |
| C2372111 | does not play with others | C2372111 |
| C2372111 | never playing with other children | C2372111 |
| C2372111 | not playing interactively | C2372111 |
| C2372111 | inability to play with other children | C2372111 |
| C2372111 | won't play with other kids | C2372111 |
| C2372111 | zero interactive play | C2372111 |
| C2372111 | no clear history of interactive play with other children | C2372111 |
| C2372111 | does not engage in reciprocal play | C2372111 |
| C2372111 | doesn't play games with family members or friends | C2372111 |
| C2372111 | no interest in playing with others | C2372111 |
| C2372111 | uninterested in playing with other children | C2372111 |
| C2372111 | no effort to initiate play with other children | C2372111 |
| C2372111 | does not usually play with other children | C2372111 |
| C2372111 | only rarely likes to play with other children | C2372111 |
| C2372111 | she does engage in interactive play with other children easily | C2372111 |
| C2372111 | he engages in little cooperative play | C2372111 |
| C2372111 | little in the way of social interactive play | C2372111 |
| C2372111 | does not engage or play with other children normally | C2372111 |
| C2372111 | difficulty playing with peers, particularly in terms of both initiating and maintaining contact | C2372111 |
| C2372111 | engages in no definite social play with age mates | C2372111 |
| C2372111 | no effort to initiate play with other children | C2372111 |
| C2372111 | did not participate in any turn taking activities | C2372111 |
| C2372111 | avoidant of mutual play | C2372111 |
| C2372111 | avoids play with his peers | C2372111 |
| C2372111 | solitary play | C2372111 |
| C2372113 | parallel play | C2372113 |
| C2372113 | parallel play only | C2372113 |
| C2372113 | prefers parallel play | C2372113 |
| C2372113 | likes to be around children, doesn't play with them | C2372113 |
| C2372113 | observes other children rather than join in | C2372113 |
| C2372113 | mostly watches other children | C2372113 |
| C2372113 | watches children, but doesn't participate | C2372113 |
| C2372113 | seems to want to be in the same room as other children, but is not actively involved in their activity | C2372113 |
| C1287531 | interest in peers | C1287531 |
| C1287531 | interested in interacting with other children | C1287531 |
| C1287531 | interested in interacting with family or other children | C1287531 |
| C1287531 | shows interest in peer relations | C1287531 |
| -C1287531 | no interest in peers | C1287531 |
| -C1287531 | not interested in interacting with other children | C1287531 |
| -C1287531 | uninterested in interacting with family or other children | C1287531 |
| -C1287531 | does not show any interest in peer relations | C1287531 |
| C0150080 | socially isolated | C0150080 |
| C0150080 | socially, he isolates himself | C0150080 |
| C0150080 | isolative behavior | C0150080 |
| C0150080 | loner | C0150080 |
| C0150080 | always been a loner | C0150080 |
| C0150080 | parents describe him as a loner | C0150080 |
| C0150080 | socially, he is a loner | C0150080 |
| C0150080 | pretty much a loner | C0150080 |
| C0150080 | no friends | C0150080 |
| C0150080 | no friends in his peer group | C0150080 |
| C0150080 | prefers isolated/autonomous activities; no close friends | C0150080 |
| C0150080 | has made few if any friends | C0150080 |
| C0150080 | impaired social interaction | C0150080 |
| C0150080 | impaired social interactions | C0150080 |
| C0150080 | social communication disorder | C0150080 |
| C0150080 | social interaction disorder | C0150080 |
| C2675043 | decreased interaction with other children | C2675043 |
| C2675043 | does not interact much with other children | C2675043 |
| C2675043 | doesn't have much interaction with children | C2675043 |
| C2675043 | limited in interactions with other children | C2675043 |
| C2675043 | minimal interaction with peers | C2675043 |
| C2675043 | impairment of peer relationships | C2675043 |
| C2675043 | does not have good peer relationships | C2675043 |
| C2675043 | history of failure to develop peer relationships | C2675043 |
| C2675043 | does not mix with other children | C2675043 |
| C2675043 | has great difficulty interacting with other children | C2675043 |
| C2675043 | does not engage or play with other children normally | C2675043 |
| C2675043 | lacks socialization skills with other children | C2675043 |
| C2675043 | very limited in the quality of social interaction with other children | C2675043 |
| C2675043 | poor social skills or interest in social interaction with peers | C2675043 |
| C2675043 | has friends, but is socially inept | C2675043 |
| C2675043 | differently interacting with other children | C2675043 |
| C2675043 | continues to manifest atypicalities in interactional style and language pattern | C2675043 |
| C2675043 | limited social interaction | C2675043 |
| C2675043 | very little interaction with people around him | C2675043 |
| C2675043 | his interactions with others tends to be quite limited | C2675043 |
| C2675043 | limited interaction with other individuals | C2675043 |
| C2675043 | lost any interest in playing interactive games | C2675043 |
| C2675043 | very little in the way of social responsiveness or rapport | C2675043 |
| C2675043 | restricted ability to initiate contact | C2675043 |
| C2675043 | not socially connected to parents | C2675043 |
| C2675043 | consistent lack of social reciprocity | C2675043 |
| C2675043 | withdrawn, passive behavior | C2675043 |
| C2675043 | peculiar, avoidant, disengaged | C2675043 |
| C2675043 | social withdrawal | C2675043 |
| C2675043 | not engaging socially | C2675043 |
| C2675043 | cannot be engaged | C2675043 |
| C2675043 | not really connected socially | C2675043 |
| C2675043 | no social responsiveness | C2675043 |
| C2675043 | refused efforts to interact | C2675043 |
| C2675043 | no attempt to interact with examiner | C2675043 |
| C2675043 | not attending to social approaches from adults | C2675043 |
| C2675043 | he will resist being held, hugged or shown physical affection | C2675043 |
| C2675043 | totally unresponsive to efforts to engage him interpersonally | C2675043 |
| C2675043 | did not actively engage with this examiner | C2675043 |
| C2675043 | difficulty with social interaction | C2675043 |
| C2675043 | disregards all attempts to interact with him | C2675043 |
| C2675043 | could not be engaged in social interaction | C2675043 |
| C2675043 | socially disconnected | C2675043 |
| C2675043 | relatively poor social interaction | C2675043 |
| C2675043 | will not interact in a socially meaningful manner | C2675043 |
| C2675043 | general disregard in social interaction | C2675043 |
| C2675043 | minimal interaction with examiner | C2675043 |
| C2675043 | clear-cut abnormalities in area of social interaction | C2675043 |
| C2675043 | pays little attention to family or examiner | C2675043 |
| C2675043 | decreased social interactions | C2675043 |
| C2675043 | limited interaction with other individuals | C2675043 |
| C2675043 | his interactions with others tends to be quite limited | C2675043 |
| C2675043 | social interaction limited, particularly in group setting | C2675043 |
| C2675043 | severe delay in socializations | C2675043 |
| C2675043 | little social interaction with others | C2675043 |
| C2675043 | social disconnectedness | C2675043 |
| C2675043 | social interaction very limited | C2675043 |
| C2675043 | social interaction clearly disturbed | C2675043 |
| C2675043 | marked impairment of interpersonal relations | C2675043 |
| C2675043 | problems with reciprocal social interactions | C2675043 |
| C2675043 | difficulty establishing close relationships | C2675043 |
| C2675043 | lacks social skills (poor, impaired, limited) | C2675043 |
| C2675043 | resists being held, hugged or shown physical affection | C2675043 |
| C2675043 | atypical child with whom to interact | C2675043 |
| -C2372078 | has to be drawn into interactions with other children | C2372078 |
| -C2372078 | peer interactions are directed by other children | C2372078 |
| -C2372078 | does not seek out contact with other children | C2372078 |
| -C2372078 | did not initiate any contact | C2372078 |
| -C2372078 | she is not seeking social interactions | C2372078 |
| -C2372078 | she certainly does not seek out attention | C2372078 |
| -C2372078 | no spontaneous social interaction | C2372078 |
| -C2372078 | he does not spontaneously seek to share interests or achievements with other people | C2372078 |
| -C2372078 | did not show any evidence of initiating an interaction | C2372078 |
| -C2372078 | initiates only limited social exchanges | C2372078 |
| -C2372078 | initiates little in the way of contact with friends | C2372078 |
| -C2372078 | initiates very little in the way of play behavior | C2372078 |
| -C2372078 | does not initiate any social behavior with family or individuals | C2372078 |
| -C2372078 | still not able to initiate a social interaction | C2372078 |
| -C2372078 | not spontaneously engaging in other children | C2372078 |
| -C2372078 | no voluntary interaction with therapist | C2372078 |
| -C2372078 | did not initiate any interaction, verbal or nonverbal | C2372078 |
| C2372078 | enters into interactions with other children | C2372078 |
| C2372078 | peer interactions are directed by self | C2372078 |
| C2372078 | seeks out contact with other children | C2372078 |
| C2372078 | initiating social interactions | C2372078 |
| C2372078 | did initiate contact | C2372078 |
| C2372078 | is seeking social interactions | C2372078 |
| C2372078 | certainly does seek out attention | C2372078 |
| C2372078 | spontaneous social interaction | C2372078 |
| C2372078 | does spontaneously seek to share interests or achievements with other people | C2372078 |
| C2372078 | did show evidence of initiating an interaction | C2372078 |
| C2372078 | initiates social exchanges | C2372078 |
| C2372078 | initiates contact with friends | C2372078 |
| C2372078 | initiates play behavior | C2372078 |
| C2372078 | initiates social behavior with family or individuals | C2372078 |
| C2372078 | able to initiate a social interaction | C2372078 |
| C2372078 | spontaneously engaging in other children | C2372078 |
| C2372078 | voluntary interaction with therapist | C2372078 |
| C2372078 | initiate any interaction, verbal or nonverbal | C2372078 |
| C0871226 | lack of "connecting" | C0871226 |
| C0871226 | talking to self | C0871226 |
| C0871226 | played with toys by mother's side | C0871226 |
| C0871226 | self-directed behavior | C0871226 |
| C0871226 | self-directed | C0871226 |
| C0871226 | reciprocity | C0871226 |
| ^£^CHIP1000201 | plays with people as objects | CHIP1000201 |
| CHIP1000201 | uses hand as tool | CHIP1000201 |
| CHIP1000201 | uses examiner's hand as tool | CHIP1000201 |
| CHIP1000201 | uses people as objects | CHIP1000201 |
| -C1822049 | does not interact with other children | C1822049 |
| -C1822049 | does not interact with peers | C1822049 |
| -C1822049 | she is not interactive | C1822049 |
| -C1822049 | refusal to interact with other children | C1822049 |
| -C1822049 | zero interaction with people | C1822049 |
| -C1822049 | doesn't socially interact | C1822049 |
| -C1822049 | uninterested in interacting with family or other children | C1822049 |
| -C1822049 | not interacting socially with others | C1822049 |
| -C1822049 | he has no social interaction with other children | C1822049 |
| -C1822049 | does not engage in meaningful reciprocal social interactions | C1822049 |
| C1822049 | interacts with other children | C1822049 |
| C1822049 | interacts with peers | C1822049 |
| C1822049 | is interactive | C1822049 |
| C1822049 | agrees to interact with other children | C1822049 |
| C1822049 | interaction with people | C1822049 |
| C1822049 | socially interacts | C1822049 |
| C1822049 | interested in interacting with family or other children | C1822049 |
| C1822049 | interacting socially with others | C1822049 |
| C1822049 | has social interaction with other children | C1822049 |
| C1822049 | engages in meaningful reciprocal social interactions | C1822049 |
| C0424095 | looking through you, content to be in her own little world | C0424095 |
| C0424095 | mostly ignored me | C0424095 |
| C0424095 | ignoring questions | C0424095 |
| C0424095 | is in his own little world | C0424095 |
| C0424095 | behaves as if deaf | C0424095 |
| C0424095 | unresponsive to requests | C0424095 |
| C0424095 | starring blankly into space | C0424095 |
| C0424095 | interacted with examiner in pleasant, but disengaged, manner | C0424095 |
| C0424095 | sense of disconnectedness was frequently present | C0424095 |
| C0424095 | interacted with examiner in quiet, withdrawn manner | C0424095 |
| C0424095 | somewhat withdrawn | C0424095 |
| C0424095 | social withdrawal | C0424095 |
| C0424095 | withdrawal from social contact | C0424095 |
| C0424095 | withdrawal; social | C0424095 |
| C0023012 | delay language | C0023012 |
| C0023012 | language delay | C0023012 |
| C0023012 | language delays | C0023012 |
| C0023012 | delayed language abilities | C0023012 |
| C0023012 | profound language delay | C0023012 |
| C0023012 | has had profound language delay | C0023012 |
| C0023012 | language milestones quite delayed | C0023012 |
| C0023012 | language has always been delayed | C0023012 |
| C0023012 | language skills limited | C0023012 |
| C0023012 | marked impairment in language | C0023012 |
| C0023012 | has some language difficulties as well as flipping words in sentences and difficulty with pronunciation | C0023012 |
| C0023012 | delayed or lack of receptive language | C0023012 |
| C0023012 | receptive & expressive language difficulties | C0023012 |
| C0023012 | delayed language | C0023012 |
| C0023012 | delayed; language | C0023012 |
| C0023012 | language delayed | C0023012 |
| C0241210 | speech delay | C0241210 |
| C0241210 | overall speech is delayed | C0241210 |
| C0241210 | limited speech | C0241210 |
| C0241210 | delayed speech | C0241210 |
| C0241210 | delays speech | C0241210 |
| C0241210 | speech delayed | C0241210 |
| C0241210 | speech delays | C0241210 |
| C0241210 | delayed articulation | C0241210 |
| C0241210 | delayed; articulation | C0241210 |
| C0241210 | delayed; speech | C0241210 |
| C0241210 | slow to talk | C0241210 |
| C1864999 | delayed speech and language development | C1864999 |
| C1864999 | significantly delayed speech and language development | C1864999 |
| C1864999 | severe speech/language retardation | C1864999 |
| C1864999 | speech and language delay | C1864999 |
| C1864999 | delayed speech and language | C1864999 |
| -C150868 | non-communicating child | C150868 |
| -C150868 | he does not talk | C150868 |
| -C150868 | no verbal communication | C150868 |
| -C150868 | no conversational speech | C150868 |
| -C150868 | no intelligible speech | C150868 |
| -C150868 | language non-existent | C150868 |
| -C150868 | no functional language | C150868 |
| -C150868 | no meaningful language | C150868 |
| -C150868 | lack of expressive language | C150868 |
| -C150868 | never developed normal speech or language | C150868 |
| -C150868 | communicates by grunting, screaming, crying | C150868 |
| -C150868 | non-verbal | C150868 |
| -C150868 | use of jargon | C150868 |
| C01508020 | verbal communication | C01508020 |
| C01508020 | communication verbal | C01508020 |
| C0746940 | no words | C0746940 |
| C0746940 | she has no words | C0746940 |
| C0746940 | nonverbal | C0746940 |
| C2673260 | has about five words | C2673260 |
| C2673260 | language consists of "Barney" and " no" | C2673260 |
| C2673260 | rarely says any words | C2673260 |
| C2673260 | limited expressive language | C2673260 |
| C0581878 | uses only single words | C0581878 |
| C0581878 | unable to speak in sentences | C0581878 |
| C0581878 | he has very little in the way of sentences | C0581878 |
| C0581878 | string 2 to 3 words together at a time | C0581878 |
| C0581878 | spoke in broken sentences ("me do") | C0581878 |
| C0581878 | very little verbal response | C0581878 |
| C0581878 | limited vocabulary | C0581878 |
| C0581878 | speech limited | C0581878 |
| C0454622 | Difficulty conveying wants & needs with language | C0454622 |
| C0454622 | Unable to ask for things using words | C0454622 |
| C0454622 | Difficulty answering questions | C0454622 |
| C0454622 | atypical pragmatic language skills | C0454622 |
| C0454622 | social use of language was below expectations | C0454622 |
| C0454622 | moderate impairment in pragmatic language | C0454622 |
| C0454622 | fails to use language as a form of social interaction | C0454622 |
| C0454622 | difficulty with concrete speech and language | C0454622 |
| C0454622 | tends to communicate by grunting & screaming | C0454622 |
| C0454622 | his understanding of pragmatic language appears to be quite limited | C0454622 |
| C0454622 | answers virtually no questions in a meaningful manner | C0454622 |
| C0454622 | significant lack in communication skills | C0454622 |
| C0454622 | does not appropriately respond verbally | C0454622 |
| C0454622 | semantic-pragmatic impairment | C0454622 |
| C0566090 | unable to sustain conversation | C0566090 |
| C0566090 | difficulty sustaining conversation | C0566090 |
| C0566090 | unable to maintain conversation | C0566090 |
| C0566068 | does not initiate conversation | C0566068 |
| C0566068 | cannot initiate conversation | C0566068 |
| C0566068 | lack of communicator initiative & responsiveness | C0566068 |
| C0566063 | difficult to engage in spontaneous conversation | C0566063 |
| C0566063 | appears not to pick up on conversational cues well | C0566063 |
| C0566063 | difficulty with conversational skills | C0566063 |
| C0566063 | no functional language | C0566063 |
| C0566063 | no pragmatic language | C0566063 |
| C0566063 | content of speech seems lacking much of the time | C0566063 |
| C0566063 | no spontaneous speech | C0566063 |
| C0566063 | difficulty making conversation | C0566063 |
| C0013528 | Echolalia | C0013528 |
| C0013528 | speech echolalic | C0013528 |
| C0013528 | echolalia tendencies heard | C0013528 |
| C0013528 | great deal of echolalia | C0013528 |
| C0013528 | delayed echolalia | C0013528 |
| C0013528 | occasionally echolalic | C0013528 |
| C0013528 | some echolalia | C0013528 |
| C0013528 | some evidence of echolalia | C0013528 |
| C0013528 | some echolalic tendencies were heard | C0013528 |
| C0013528 | echolalic tendencies in his toddler years | C0013528 |
| C0013528 | language continues to be quite stereotypical and quite echolalic | C0013528 |
| C0013528 | expressive language, often echolalic-like | C0013528 |
| C0013528 | seems to parrot or mimic what is said | C0013528 |
| C0013528 | echo speech | C0013528 |
| C0013528 | echo reaction | C0013528 |
| C0013528 | echophrasia | C0013528 |
| C0013528 | reaction, echo | C0013528 |
| C0013528 | speech, echo | C0013528 |
| C0392185 | repeating words | C0392185 |
| C0392185 | repeated certain words over and over | C0392185 |
| C0392185 | much of his speech is repetitive | C0392185 |
| C0392185 | repetitive, stereotypic utterances | C0392185 |
| C0392185 | repetitious, recitation-like quality | C0392185 |
| C0392185 | repetitive stereotypical phrases such as 'you bet' | C0392185 |
| C0392185 | repeating sounds in perseverative way | C0392185 |
| C0392185 | often perseverated with a word for days at a time | C0392185 |
| C0392185 | often repeats phrases from videos | C0392185 |
| C0392185 | repeats / imitates almost all words said to him | C0392185 |
| C0392185 | some self-stimulating verbal activity - stereotype behavior of naming letters & some numbers with articulation | C0392185 |
| C0392185 | verbal repetition | C0392185 |
| C0392185 | repetitive speech | C0392185 |
| C0392185 | palilalia | C0392185 |
| C0392185 | repetition, NOS | C0392185 |
| C0514261 | speaking in third person | C0514261 |
| C0514261 | difficulty understanding appropriate use of pronouns and speaking | C0514261 |
| C0514261 | does not use pronouns | C0514261 |
| CHIP1000204 | stereotypical utterances | CHIP1000204 |
| CHIP1000204 | stereotypic vocalization | CHIP1000204 |
| CHIP1000204 | stereotyped phrases | CHIP1000204 |
| CHIP1000204 | rote phrases | CHIP1000204 |
| C0233726 | mimics sounds | C0233726 |
| C0233726 | sing-songy sounds | C0233726 |
| C0233726 | making voices | C0233726 |
| C0233726 | atypical prosody | C0233726 |
| C0233726 | aprosodia | C0233726 |
| C0233726 | aprosodic speech | C0233726 |
| C0233726 | speech, aprosodic | C0233726 |
| C0233726 | aprosody | C0233726 |
| C2018115 | high-pitched and repetitive vocalizations | C2018115 |
| C2018115 | high pitched quality of speech | C2018115 |
| C2018115 | speech often shrill / high pitched when excited | C2018115 |
| C2018115 | prosodic contour odd, generally high in pitch | C2018115 |
| C2018115 | speech tone high-pitched | C2018115 |
| C2674583 | idiosyncratic language abnormalities | C2674583 |
| C2674583 | talks / answers himself in a different voice | C2674583 |
| C2674583 | unusual noises | C2674583 |
| C2674583 | making hee-haw sounds | C2674583 |
| C2674583 | strange, unusual 'oowing' noise | C2674583 |
| C2674583 | unusual sounds such as squealing or guttural sounds | C2674583 |
| C2674583 | auditory self-stimulation - singing, vocalizations | C2674583 |
| C2674583 | repeating non-sense sounds | C2674583 |
| C2674583 | inappropriate giggling | C2674583 |
| C2674583 | unintelligible speech | C2674583 |
| C2674583 | unintelligible vocalizations | C2674583 |
| C2674583 | grunting | C2674583 |
| -C517121 | stopped using toys | C517121 |
| -C517121 | does not play with toys | C517121 |
| -C517121 | does not play with manipulative toys | C517121 |
| -C517121 | no meaningful interaction with toys | C517121 |
| -C517121 | not engaging in any specific play activity | C517121 |
| -C517121 | inspected toys but did not play with toys | C517121 |
| C0517073 | started using toys | C0517073 |
| C0517073 | plays with toys | C0517073 |
| C0517073 | plays with manipulative toys | C0517073 |
| C0517073 | meaningful interaction with toys | C0517073 |
| C0517073 | engaging in specific play activity | C0517073 |
| C0517073 | inspected toys and played with toys | C0517073 |
| C1822065 | no imaginative play | C1822065 |
| C1822065 | play with toys was without imaginative control | C1822065 |
| C1822065 | no evidence of creative play | C1822065 |
| C1822065 | no indication of creative or representational play | C1822065 |
| C1822065 | little evidence of pretend / creative play | C1822065 |
| C1822065 | he is not involved in interactive play | C1822065 |
| C1822065 | she did not engage in my obvious pretend play | C1822065 |
| C1822065 | very significant lack of interest in make-believe play | C1822065 |
| C1822065 | lacks spontaneous make believe play | C1822065 |
| C1822065 | no symbolic play | C1822065 |
| C1822065 | engages in creative play | C1822065 |
| -C517121 | does not play with toys appropriately | C517121 |
| -C517121 | toys not used in any purposeful manner | C517121 |
| -C517121 | interacted with toys only by naming them | C517121 |
| C0517073 | plays with toys appropriately | C0517073 |
| C0517073 | toys used in purposeful manner | C0517073 |
| C0517073 | interacted with toys in addition to naming them | C0517073 |
| C0517073 | shows interest in toys | C0517073 |
| -C1822065 | imaginary play minimal | C1822065 |
| -C1822065 | does not engage in much imaginative play | C1822065 |
| C1822065 | imaginary play great | C1822065 |
| C1822065 | engages in much imaginative play | C1822065 |
| C1822065 | engages in creative play | C1822065 |
| -C2584305 | No imitative play | C2584305 |
| -C2584305 | nothing to suggest imitation | C2584305 |
| C2584305 | imitative play | C2584305 |
| C2584305 | suggests imitation | C2584305 |
| C2584305 | copying | C2584305 |
| C2584305 | imitation | C2584305 |
| C2584305 | learning imitation | C2584305 |
| C2584305 | copying (learning) | C2584305 |
| C2584305 | imitation (learning) | C2584305 |
| C0424268 | lining up toys | C0424268 |
| C0424268 | repetitively lining up toys | C0424268 |
| C0424268 | lining up cars | C0424268 |
| C0424268 | lines up objects | C0424268 |
| C0424268 | likes to line objects up | C0424268 |
| C0424268 | only plays by lining things up | C0424268 |
| C0424268 | arranging objects in straight line | C0424268 |
| -C2372110 | limited play | C2372110 |
| -C2372110 | play is limited and quite stereotypic | C2372110 |
| -C2372110 | play is extremely limited | C2372110 |
| -C2372110 | play was simple and repetitive | C2372110 |
| -C2372110 | continues stereotypical play | C2372110 |
| -C2372110 | perseverative play | C2372110 |
| -C2372110 | play was simple and repetitive, indeed perseverative | C2372110 |
| -C2372110 | trouble playing with toys | C2372110 |
| -C2372110 | shows little interest in toys | C2372110 |
| -C2372110 | narrowed involvement with play activities | C2372110 |
| -C2372110 | play with toys is not very functional | C2372110 |
| -C2372110 | plays randomly with toys | C2372110 |
| -C2372110 | has few toys | C2372110 |
| -C2372110 | only a few toys with which he will play | C2372110 |
| -C2372110 | play is limited to a few preferred items | C2372110 |
| -C2372110 | does not play with toys in the fashion intended | C2372110 |
| -C2372110 | plays abnormally with toys | C2372110 |
| -C2372110 | will repeatedly toss blocks to floor and watch them scatter | C2372110 |
| C2675334 | restrictive behavior, interests, and activities | C2675334 |
| C2675334 | fascination with water | C2675334 |
| C2675334 | preoccupation with water | C2675334 |
| C2675334 | repetitively playing with water | C2675334 |
| C2675334 | profound fascination for water, balloon | C2675334 |
| C2675334 | memorizing telephone numbers | C2675334 |
| C2675334 | memorizes lines and dialogue from movies and stories | C2675334 |
| C2675334 | memorization of lengthy book passages | C2675334 |
| C2675334 | perseverates on various topics | C2675334 |
| C2675334 | perseverates on a single topic | C2675334 |
| C0562463 | fascination with spinning fans | C0562463 |
| C0562463 | enjoys watching spinning fans | C0562463 |
| C0562463 | fascinated by spinning objects | C0562463 |
| C0562463 | likes to watch objects spin | C0562463 |
| C0562463 | turning / twirling objects | C0562463 |
| C0562463 | repetitive spinning of objects | C0562463 |
| C2114104 | Preoccupation with fire | C2114104 |
| C2114104 | preoccupied with medical instruments | C2114104 |
| C2114104 | fascinated by the sound when computer is turned on; when air is squeezed out of bottle | C2114104 |
| C2114104 | peculiar preoccupation with horizontal and vertical planes | C2114104 |
| C2114104 | preoccupation with bouncing ball | C2114104 |
| C2114104 | likes to watch the mixer | C2114104 |
| C2114104 | very preoccupied with cartoons | C2114104 |
| C2114104 | fixated on horses | C2114104 |
| C2114104 | fascinated by mechanical objects | C2114104 |
| C2114104 | trains | C2114104 |
| C2114104 | obsessed with elevators, writing, and drawing | C2114104 |
| C2114104 | obsesses on bendable objects | C2114104 |
| C2114104 | fascinated by anything with letters | C2114104 |
| C2114104 | grasshoppers | C2114104 |
| C2114104 | obsessively focused on books, videos, and pictures | C2114104 |
| C2114104 | preoccupation with cutting up paper into small pieces | C2114104 |
| C2114104 | preoccupied with wires and cords | C2114104 |
| C2114104 | preoccupation with trash, bathrooms, memorizing birthdays | C2114104 |
| C2114104 | fascination with engines and keys | C2114104 |
| C2114104 | tipping a cup of water into the sink over and over | C2114104 |
| C2114104 | repeatedly putting puzzles together | C2114104 |
| C2114104 | compulsion for jigsaw puzzles | C2114104 |
| C2114104 | enamored of television country music station, will watch for 2-3 hours (note: issue is NOT time it's the 'enamored/perseverative' issue) | C2114104 |
| C2114104 | fixation on certain objects and colors | C2114104 |
| C2114104 | lining up objects | C2114104 |
| C2114104 | repetitive activities such as counting objects | C2114104 |
| C2114104 | obsessed with tire swings, color yellow (stacking only yellow blocks, using only yellow crayons) | C2114104 |
| C2114104 | preoccupied with living things | C2114104 |
| C2114104 | preoccupation of patterns of doing things | C2114104 |
| C2114104 | restricted interest in play | C2114104 |
| C2114104 | playing excessively with certain objects | C2114104 |
| C2114104 | excessive focus on cars and trains | C2114104 |
| C2114104 | spends much time filtering corn through his hands | C2114104 |
| C2114104 | she will stare at books or wrap string around fingers for hours | C2114104 |
| C2114104 | playing with cage / netting over and over | C2114104 |
| C2114104 | fans the pages of a book 20 minutes at a time | C2114104 |
| C2114104 | staring into mirror, lights | C2114104 |
| C2114104 | has watched particular video 100-200 times | C2114104 |
| C2114104 | likes to do puzzles over and over again | C2114104 |
| C2114104 | play on trampoline for hours | C2114104 |
| C2114104 | picking at wallpaper until whole room is free of wallpaper | C2114104 |
| C2114104 | he arranges his cars over and over again in lines | C2114104 |
| C2114104 | playing for long periods with trains, arranging and rearranging in different patterns | C2114104 |
| C2114104 | his favorite activity is to walk around waving coat hangers in front of himself | C2114104 |
| C2114104 | fills and empties bank with coins repeatedly | C2114104 |
| C2114104 | likes to tie things together | C2114104 |
| C2114104 | likes playing with boxes, will line them up in a stereotypical manner and will walk around and look at them | C2114104 |
| C2114104 | his play is described as intense | C2114104 |
| C2114104 | he can become fixated on an activity to the point that it is impossible to redirect him | C2114104 |
| C2114104 | very focused and intense on single activities / objects to the point of tuning everyone out | C2114104 |
| C2114104 | preoccupation with television | C2114104 |
| C2114104 | he can be totally absorbed in watching television | C2114104 |
| C2114104 | perseverative at watching videos and other activities | C2114104 |
| C2114104 | preoccupation with narrowly focused interest | C2114104 |
| C0475222 | ritualized behaviors | C0475222 |
| C0475222 | needing to get out of car in certain way, touching walls in rooms | C0475222 |
| C0475222 | need to touch walls in certain places at certain times of the day | C0475222 |
| C0475222 | smells everything | C0475222 |
| C0475222 | stacking books, touching rituals | C0475222 |
| C0475222 | number of rituals | C0475222 |
| C0475222 | preoccupied with touching things | C0475222 |
| C0475222 | walks in straight and specific patterns | C0475222 |
| C0475222 | obsessive rituals before going to bed, opening cupboards | C0475222 |
| C0475222 | requires that his brother say exact same words each night during the bedtime ritual | C0475222 |
| C0475222 | fast forward and rewinds videos to which he is deeply attached and sleeps with | C0475222 |
| C0475222 | licking carpet during exam | C0475222 |
| C0475222 | ritualistic behavior | C0475222 |
| C2675335 | Lining up toys | C2675335 |
| C2675335 | lining up, grouping toys | C2675335 |
| C2675335 | if this activity is disrupted (lining up objects), he exhibits anger | C2675335 |
| C2675335 | lining up pictures of presidents | C2675335 |
| C2675335 | tends to twirl ropes or strings throughout the house repetitively | C2675335 |
| C2675335 | setting up, knocking down objects repeatedly | C2675335 |
| C2675335 | turning light switch on and off repeatedly | C2675335 |
| C2675335 | turning water and light switches on and off | C2675335 |
| C2675335 | engaging in some repetitive behaviors | C2675335 |
| C2675335 | perseverative behaviors | C2675335 |
| C2675335 | repetitive hand washing | C2675335 |
| C2675335 | engaging in some repetitive behavior | C2675335 |
| C2675335 | repetitious behavior | C2675335 |
| C2675335 | repetitively labeling objects | C2675335 |
| C2675335 | likes to have something in hand which he tends to fondle and tear up | C2675335 |
| C2675335 | stereotyped, repetitive behavior | C2675335 |
| C1837653 | resistant to change | C1837653 |
| C1837653 | usually resists undertaking any activity | C1837653 |
| C1837653 | likes repetition and routine | C1837653 |
| C1837653 | shows a need for routine | C1837653 |
| C1837653 | inflexibility with regard to routines | C1837653 |
| C1837653 | difficulty with changes in routine | C1837653 |
| C1837653 | rigidity of routines | C1837653 |
| C1837653 | likes to have matters on things the same | C1837653 |
| C1837653 | makes effort at maintaining routine | C1837653 |
| C1837653 | insists on sameness | C1837653 |
| C1837653 | insistence on sameness | C1837653 |
| C1837653 | desire for sameness, some intolerance for change | C1837653 |
| C1837653 | very demanding of sameness | C1837653 |
| C1837653 | he seems very fixated on doing things in his own order | C1837653 |
| C1837653 | he cannot tolerate changes in his routine | C1837653 |
| C1837653 | quite marked resistance to change | C1837653 |
| C1837653 | becomes upset if parents fail to follow specific car route | C1837653 |
| C1837653 | distress if parents drive different way to grandmother's house | C1837653 |
| C1837653 | routines very important; if altered he becomes agitated | C1837653 |
| C1837653 | changes can be very upsetting to her | C1837653 |
| C1837653 | prefers a consistent routine; cries if changes are made | C1837653 |
| C1837653 | easily upset when routines are broken | C1837653 |
| C1837653 | easily upset by change in routines | C1837653 |
| C1837653 | upset if meals don't occur at exactly the same time | C1837653 |
| C1837653 | angry in response to change in routine | C1837653 |
| C1837653 | becomes very upset with change to his routine | C1837653 |
| C1837653 | if the routines of her life are broken, she becomes quite angry | C1837653 |
| C1837653 | refusing to eat food if not cut in a certain way | C1837653 |
| C1837653 | needs lots of structure and consistency | C1837653 |
| C1837653 | fearful to try new activities | C1837653 |
| C1837653 | adherence to routine | C1837653 |
| C1837653 | inflexible adherence to routines or rituals | C1837653 |
| C0424247 | Hand flapping | C0424247 |
| C0424247 | hand flapping when excited | C0424247 |
| C0424247 | hand flapping when agitated | C0424247 |
| C0424247 | arm waving when excited | C0424247 |
| C0424247 | flapping movements of hands | C0424247 |
| C1853236 | Hand movements | C1853236 |
| C1853236 | hand waving | C1853236 |
| C1853236 | hand shaking | C1853236 |
| C1853236 | unusual hand movements | C1853236 |
| C1853236 | repeatedly turning over hands | C1853236 |
| C1853236 | shaking of hands, shuddering | C1853236 |
| C1853236 | clapping hands on thighs in a very rapid fashion | C1853236 |
| C1853236 | finger wiggling | C1853236 |
| C1853236 | hand / finger wiggling | C1853236 |
| C1853236 | hand clapping | C1853236 |
| C1853236 | repetitive clapping | C1853236 |
| C1853236 | number of instances of idiosyncratic movements with his hands | C1853236 |
| C1853236 | idiosyncratic movements, walking fingers across table | C1853236 |
| C1853236 | stereotypic finger movements | C1853236 |
| C1853236 | repetitive finger movements | C1853236 |
| C1853236 | repetitive flexion / extension movements of his fingers | C1853236 |
| C1853236 | loves to tap finger on table | C1853236 |
| C1853236 | tapping finger and thumb together | C1853236 |
| C1853236 | rapid finger movements | C1853236 |
| C1853236 | finger flicking | C1853236 |
| C1853236 | finger flicking in front of face | C1853236 |
| C1853236 | waving things in front of his face | C1853236 |
| C1853236 | some finger waving by eye | C1853236 |
| C1853236 | finger-play behavior | C1853236 |
| C1853236 | ringing of hands | C1853236 |
| C1853236 | stereotypic movements | C1853236 |
| C1853236 | stereotypical movements | C1853236 |
| C1853236 | Stereotypical motor behavior | C1853236 |
| C1853236 | atypical stereotypic motor mannerisms | C1853236 |
| C1853236 | some stereotypic movements observed | C1853236 |
| C1853236 | some self-stimulatory behavior | C1853236 |
| C1853236 | some stereotypic hand movements were observed | C1853236 |
| C1853236 | occasions in which he will demonstrate repetitive movements | C1853236 |
| C1853236 | repetitive mannerisms described | C1853236 |
| C1853236 | has manifested stereotypic - like movements | C1853236 |
| C1853236 | picks at nose stereotypically with thumb and index finger | C1853236 |
| C1853236 | picking at her nose for hours at a time | C1853236 |
| C1853236 | moving his jaw, rubbing his hands together | C1853236 |
| C1853236 | hitting jaw with hand to click teeth | C1853236 |
| C1853236 | picks at his skin | C1853236 |
| C1853236 | blowing on hand | C1853236 |
| C1853236 | hand licking | C1853236 |
| C1853236 | lip smacking | C1853236 |
| C1853236 | arm flapping | C1853236 |
| C1853236 | head shaking | C1853236 |
| C1853236 | twisting his nipples, repetitively striking his finger against table | C1853236 |
| C1853236 | body posturing | C1853236 |
| C1853236 | unusual posturing; jumping while holding crotch | C1853236 |
| C1853236 | raising one of his arms | C1853236 |
| C1853236 | moving foot in stereotypic manner | C1853236 |
| C1853236 | plugging ear with thumb | C1853236 |
| C1853236 | hair flicking | C1853236 |
| C1853236 | hopping | C1853236 |
| C1853236 | slapping hands under chin | C1853236 |
| C1853236 | motor jerks (stereotypical or repetitive - not a tic) | C1853236 |
| C1853236 | hair pulling | C1853236 |
| C1853236 | hand sucking | C1853236 |
| C1853236 | general self stimulation | C1853236 |
| C1853236 | manic - pacing | C1853236 |
| C0562462 | Spinning | C0562462 |
| C0562462 | spins around in circles | C0562462 |
| C0562462 | likes to spin in circles | C0562462 |
| C0562462 | enjoys spinning | C0562462 |
| C0562462 | he likes to spin and twirl | C0562462 |
| C0562462 | loves to spin in circles | C0562462 |
| C0562462 | loves to spin | C0562462 |
| C0562462 | twirling and spinning | C0562462 |
| C0562462 | practices spinning, twirling his body | C0562462 |
| C0562462 | self-stimulatory behaviors including spinning | C0562462 |
| C0562462 | repetitive spinning of whole body | C0562462 |
| C0562458 | pacing in circles | C0562458 |
| C0562458 | runs in circles | C0562458 |
| C0562458 | pacing up and down | C0562458 |
| C0562458 | pacing | C0562458 |
| C0562458 | pacing the floor | C0562458 |
| C2170460 | head banging | C2170460 |
| C2170460 | pounds head | C2170460 |
| C2170460 | head banging on floor repeatedly | C2170460 |
| C2170460 | hitting himself on head | C2170460 |
| C2170460 | slapping himself | C2170460 |
| C2170460 | repetitive head banging | C2170460 |
| C0005899 | Rocking | C0005899 |
| C0005899 | sits on bed and rocks | C0005899 |
| C0005899 | body rocking | C0005899 |
| C0005899 | body-rocking | C0005899 |
| C0005899 | repetitive rocking movements | C0005899 |
| C0427144 | Toe walking | C0427144 |
| C0427144 | runs on tip toes | C0427144 |
| C0427144 | toe-walking gait | C0427144 |
| C0427144 | toe-walking | C0427144 |
| C0427144 | walking on toes | C0427144 |
| C0427144 | abnormality of toe walking | C0427144 |
| C2114105 | stereotypic play with wheels | C2114105 |
| C2114105 | spinning wheels of toys | C2114105 |
| C2114105 | spinning wheels repetitively | C2114105 |
| C2114105 | enjoys spinning wheels of cars | C2114105 |
| C2114105 | watching wheels of trucks | C2114105 |
| C2114105 | likes to watch wheels spin | C2114105 |
| C2114105 | preoccupation with spinning wheels | C2114105 |
| C2114105 | focused attention on wheels of car | C2114105 |
| C2114105 | carefully scrutinizes parts of toys | C2114105 |
| C2114105 | fascinated by parts of objects | C2114105 |
| C2114105 | tends to relate to objects as their parts as opposed to their whole | C2114105 |
| C2114105 | preoccupation with parts of objects | C2114105 |
| C2114105 | opens and closes toy car doors and looks inside over and over | C2114105 |
| C2114105 | fascinated with dolls that have eyes that open and close | C2114105 |
| C2114105 | he has a fascination with objects | C2114105 |
| C2114105 | interest with mechanical devices | C2114105 |
| C1707798 | will manipulate things, take things apart | C1707798 |
| C1707798 | picking at objects until disassembled | C1707798 |
| C1707798 | disassembly | C1707798 |
| C1707798 | disassemble | C1707798 |
| C0085271 | bites fingers until red and swollen | C0085271 |
| C0085271 | arm biting | C0085271 |
| C0085271 | picking or pulling hair out | C0085271 |
| C0085271 | self-injurious behavior | C0085271 |
| C0085271 | deliberate self-harm | C0085271 |
| C0085271 | deliberate self harm | C0085271 |
| C0085271 | self injurious behavior | C0085271 |
| C0085271 | self-harm, deliberate | C0085271 |
| C0085271 | self-abusive behavior | C0085271 |
| C0085271 | self-destructive behavior | C0085271 |
| C0085271 | self destructive behavior | C0085271 |
| C0457044 | bothered by loud volume on radio | C0457044 |
| C0457044 | does not like to be touched | C0457044 |
| C0457044 | tactile defensiveness (finger paints, playdough, shaving cream, etc.) | C0457044 |
| C0457044 | does not respond to pain appropriately | C0457044 |
| C0457044 | sensory intolerance | C0457044 |
| C0031873 | PICA | C0031873 |
| C0031873 | craving unusual or nonfood items | C0031873 |
| C0031873 | eats abnormal objects | C0031873 |
| C0031873 | abnormal craving | C0031873 |
| C0031873 | Inappropriate eating behavior | C0031873 |
| CHIP1000209 | Hyper- or hypo-reactivity to sensory input or unusual interest in sensory aspects of environment; (such as apparent indifference to pain/heat/cold, adverse response to specific sounds or textures, excessive smelling or touching of objects, fascination with lights or spinning objects). | CHIP1000209 |
| CHIP1000209 | hyper-reactivity to sensory input | CHIP1000209 |
| C1855589 | hypersensitive to stimuli | C1855589 |
| C2163191 | cry as hypersensitive to stimulus | C2163191 |
| C0234241 | indifference to pain | C0234241 |
| C0424795 | tolerant of heat | C0424795 |
| C0344336 | tolerant of cold | C0344336 |
| C1827779 | abnormal response to sound | C1827779 |

*CUI - Concept Unique Identifiers from Unified Medical Language System

†Codes prefixed with C are CUIs.

^£^Codes prefixed with CHIP are custom concept codes for which no CUI match exists.
